# *In vivo* proteomics identifies the competence regulon and AliB oligopeptide transporter as pathogenic factors in pneumococcal meningitis

**DOI:** 10.1371/journal.ppat.1007987

**Published:** 2019-07-29

**Authors:** Frank Schmidt, Niamatullah Kakar, Tanja C. Meyer, Maren Depke, Ilias Masouris, Gerhard Burchhardt, Alejandro Gómez-Mejia, Vishnu Dhople, Leiv S. Håvarstein, Zhi Sun, Robert L. Moritz, Uwe Völker, Uwe Koedel, Sven Hammerschmidt

**Affiliations:** 1 ZIK-FunGene, Department of Functional Genomics, Interfaculty Institute for Genetics and Functional Genomics, Center for Functional Genomics of Microbes, University Medicine Greifswald, Greifswald, Germany; 2 Department of Molecular Genetics and Infection Biology, Interfaculty Institute for Genetics and Functional Genomics, Center for Functional Genomics of Microbes, University of Greifswald, Greifswald, Germany; 3 Department of Functional Genomics, Interfaculty Institute for Genetics and Functional Genomics, Center for Functional Genomics of Microbes, University Medicine of Greifswald, Greifswald, Germany; 4 Clinic Grosshadern of the Ludwig-Maximilians University of Munich, Department of Neurology, Munich, Germany; 5 Faculty of Chemistry, Biotechnology and Food Science, Norwegian University of Life Sciences, Ås, Norway; 6 Institute for Systems Biology, Seattle, WA, United States of America; University of California, San Francisco, UNITED STATES

## Abstract

*Streptococcus pneumoniae* (pneumococci) is a leading cause of severe bacterial meningitis in many countries worldwide. To characterize the repertoire of fitness and virulence factors predominantly expressed during meningitis we performed niche-specific analysis of the *in vivo* proteome in a mouse meningitis model, in which bacteria are directly inoculated into the cerebrospinal fluid (CSF) cisterna magna. We generated a comprehensive mass spectrometry (MS) spectra library enabling bacterial proteome analysis even in the presence of eukaryotic proteins. We recovered 200,000 pneumococci from CSF obtained from meningitis mice and by MS we identified 685 pneumococci proteins in samples from *in vitro* filter controls and 249 in CSF isolates. Strikingly, the regulatory two-component system ComDE and substrate-binding protein AliB of the oligopeptide transporter system were exclusively detected in pneumococci recovered from the CSF. In the mouse meningitis model, AliB-, ComDE-, or AliB-ComDE-deficiency resulted in attenuated meningeal inflammation and disease severity when compared to wild-type pneumococci indicating the crucial role of ComDE and AliB in pneumococcal meningitis. In conclusion, we show here mechanisms of pneumococcal adaptation to a defined host compartment by a proteome-based approach. Further, this study provides the basis of a promising strategy for the identification of protein antigens critical for invasive disease caused by pneumococci and other meningeal pathogens.

## Author summary

Pneumococci are one of the most common and aggressive meningitis pathogens associated with mortality rates between 10% and 30%. Due to severe complications during therapeutic intervention, prevention strategies to combat pneumococcal meningitis (PM) are preferred. The vaccines available are so far suboptimal and inefficient to prevent serious PM. Hence, deciphering the mechanisms employed by pneumococci to encounter and survive in the cerebrospinal fluid (CSF) will pave the way for the development of new antimicrobial strategies. This work used an *in vivo* proteome-based approach to identify pneumococcal proteins expressed in the CSF during acute meningitis. This strategy identified a nutrient uptake system and regulatory system to be highly expressed in the CSF and being crucial for PM. Knocking out two of the highly *in vivo* expressed proteins (AliA and ComDE) in *S*. *pneumoniae* yields to a significant increase in survival and decrease in pathogen burden of infected mice. These host compartment specific expressed pneumococcal antigens represent promising candidates for antimicrobials or protein-based vaccines.

## Introduction

Bacterial meningitis accounts for approximately 0.5% of all deaths worldwide [1] and this catapults bacterial meningitis into the list of the top ten infectious burdens around the world [2].

*Streptococcus pneumoniae* (the pneumococcus) is one of the most common and most aggressive pathogen causing meningitis. The unfavourable outcome of pneumococcal meningitis (PM) with mortality rates of 10 to 30% is mainly due to meningitis-related brain damage [3, 4]. This damage is caused by both the massive inflammatory reaction and bacterial toxins [5]. The corresponding tremendous immune response is mainly induced by bacterial cell wall components like peptidoglycan (PGN) and lipoteichoic acid (LTA). In addition, it is known that the proteinaceous toxin pneumolysin (Ply) produced by pneumococci is a crucial bacterial factor implicated in meningitis-related brain damage [6–9].

However, the inflammatory response, brain damage, and clinical course can vary between different pneumococcal serotypes, which are all positive for PGN, LTA, and Ply [10]. This observation makes it highly conceivable that additional, yet unidentified bacterial factors contribute to bacterial fitness in CSF and regulation of meningeal inflammation and/or the damage of the brain, and thus to the clinical outcome of the disease.

However, little is known about the pneumococcal fitness factors and virulence determinants required for crossing the blood-brain-barrier and survival within the CNS. PspC (also known as CbpA), Ply, PavA, and neuraminidase A (NanA) are so far the best-studied virulence factors contributing to the development of meningitis [7, 11–13]. A recent study identified PspC and the pilus adhesin RrgA as mediators of pneumococcal brain invasion by their interaction with the polymeric Ig receptor and PECAM-1 [14]. Nevertheless, PspC-, Ply-, or NanA-deficiency had no substantial impact on the disease course in an experimental model of pneumococcal meningitis, suggesting that additional microbial fitness or virulence factors make an important contribution.

Pneumococci have evolved various successful strategies to infect humans, to adapt to different host niches, and to evade host innate immune attack mechanisms [15–17]. Identification of the genes and proteins that are specifically required for each stage of the infection process will enable a new level of understanding of the mechanisms employed by pathogens to circumvent the host defence mechanisms and cause disease. Several strategies have been employed to screen *in vivo*–induced genes, and the adaptation of pneumococci to different host milieus including nasopharynx and blood has been correlated with a differential gene expression of several virulence determinants [18–24]. Although proteins are the functional key players in bacterial infection processes, a comprehensive analysis of the proteome signatures of pneumococci under infection-related has not been performed yet. Those signatures will allow gaining insights into the adaptation and physiology of the pathogenic bacteria during colonization and dissemination in humans. Up to now, just a few *in vivo* protein-profiling approaches have been applied for *S*. *pneumoniae*, which is due to limitations in mass spectrometry (MS) sensitivity and sample complexity in natural host pathogen interactions. However, in the past decade new MS instruments and methods to isolate the pathogen from its host enables monitoring of changes of the proteome during infection as already shown for *Staphylococcus aureus* [25].

Hence, the main focus of this study was to identify protein factors involved in the pathophysiology of pneumococcal meningitis in order to provide a better understanding of the underlying molecular mechanisms of the disease. We hypothesized that microbial factors that are specifically upregulated during pneumococcal growth in the cisterna of the brain play a crucial role in pneumococcal meningitis. To test this, we conducted a proteomic analysis of bacteria harvested before and 18 hours after infection from the CSF of mice. We further examined the role of two selected up-regulated proteins in the disease course and investigated the impact of the absence of these proteins by *in vitro* proteomics.

## Results

### A SpectraST library for *Streptococcus pneumoniae*

**Fig 1:**
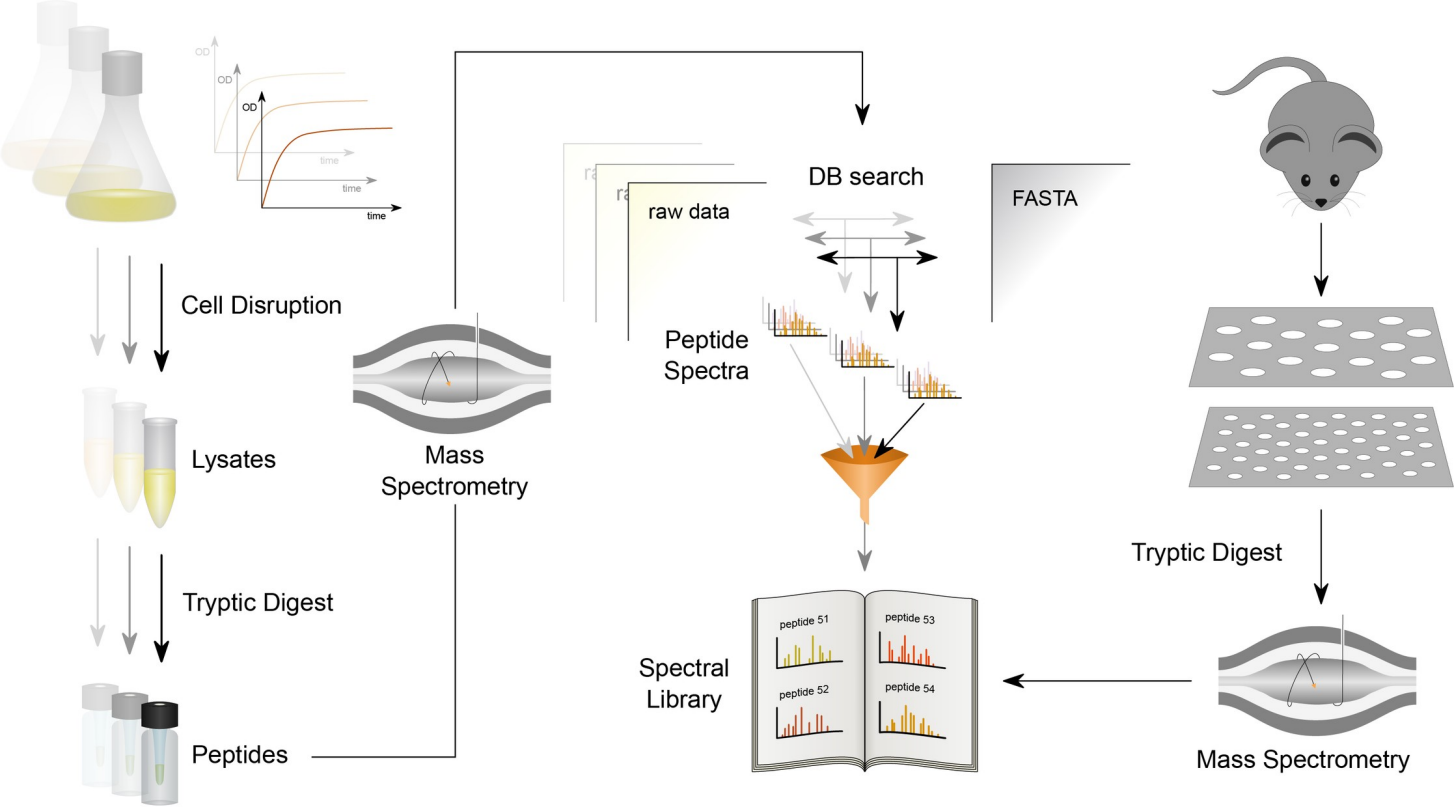
Workflow applied for the identification of pneumococcal-specific proteins from mouse CSF isolates. In a first step, *S*. *pneumoniae* D39 specific tandem-MS spectra from different conditions were measured and stored in a database (spectral library; SpectraST). Subsequently, proteins from *S*. *pneumoniae in vivo* experiments were measured after dual filter extraction and digestion steps and compared and aligned to spectra deposited in the database. A subset of proteins exclusively identified *in vivo* in the CSF was further considered for phenotypical characterization and for demonstrating its impact on pneumococcal meningitis.

The interference of different peptides within a complex mixture as represented by host-pathogen samples from infection settings is a critical step in the analysis of tandem-MS spectra. In most commonly applied data-analysis pipelines *in silico* driven putative sequences are used to identify tandem-MS spectra. However, false-discovery rate (FDR)-based identifications of these spectra are strongly depending on the ratio of naturally existing entries and *in silico* based entries. Hence, the FDR is more error-prone in case of mixed host-pathogen samples where two organisms are combined. One of the most suitable methods to bypass this limitation is the so-called spectra-to-spectra search [26]. Here the number of database entries is significantly reduced. As a prerequisite for such approaches a suitable number of tandem-MS reference spectra is necessary. High quality tandem-MS spectra from *S*. *pneumoniae* in public repositories such as Pride [27] or PeptideAtlas [28] were not available, hence, we first established a comprehensive consensus pneumococcal SpectraST library (Fig 1) [29]. We have conducted 36 measurements from 10 different conditions mimicking the physiology and natural *in vivo* milieus of *S*. *pneumoniae* (S6 Table).

**Fig 2:**
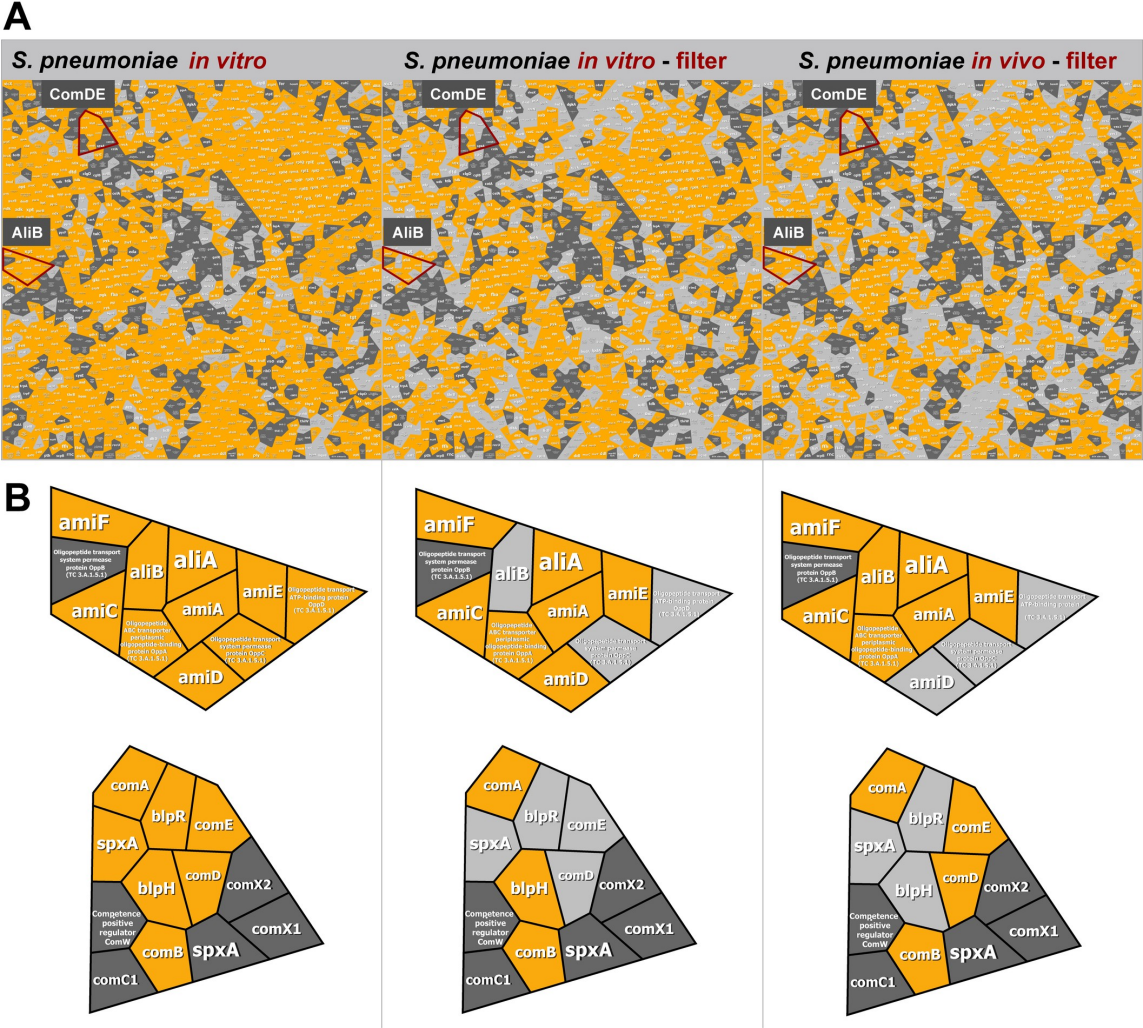
Identification of ComDE and AliB in *S*. *pneumoniae* D39 from *in vitro* culture or *in vivo* infection samples. **(A)** Voronoi Treemaps of predicted and detected pneumococcal proteins. *S*. *pneumoniae* D39 was cultured under various *in vitro* conditions, and a map of *in vitro* identified proteins was generated (left panel). Pneumococcal proteins identified in control reactions with a trypsin-digestion of pneumococci on a filter (middle panel). These pneumococci represent bacteria from a culture that was also used to infect mice. Proteins identified in pneumococci recovered from the CSF of mice (n = 5 samples each of 4 mice), enriched by sequential centrifugation on a filter, and digested by trypsin (right panel). Gray spheroids represent annotated protein entries from the SEED with light gray not identified in sample and dark gray never identified. Orange spheroids represent identified proteins by MS. **(B)** Identified proteins are depicted in enlarged regions of Voronoi treemaps. ComD, ComE (upper left), and AliB (lower left) were not identified in *S*. *pneumoniae* D39 from *in vitro* culture samples, while all three proteins were identified in samples from *in vivo* infection (upper and lower right).

Our combined analyses finally resulted in 1,165 unique protein identifications (IDs) of which 954 were further ranked above a peptide prophet threshold of 0.95 for SpectraST library construction. This correlates to approximately 70% of the annotated and categorized proteome. These proteins were represented by 49,083 tandem-MS spectra reflecting in total 7,597 unique peptides. An overview of the *in vitro* detected proteins is shown in Fig 2A.

### *In vivo* proteomics of pneumococci recovered from the CSF revealed ComDE and AliB expression during infection

One of the major challenges of host-pathogen interaction experiments is the elucidation of basic adaptation and regulation mechanisms of the infiltrating pathogen and its primary host. A large fraction of molecules that mediate host-pathogen interactions are proteins and can therefore be quantified by modern proteomics tools such as gel-free MS. In order to reduce systems complexity for proteomic screens, *in vitro* assays have been used to mimic the natural milieu of human pathogens. However, it is obvious that *in vitro* assays can only partially capture the complex interactions in murine animal experiments or human sample specimen. Thus, more efforts should be invested to develop novel and sophisticated *in vivo* proteomics approaches. Here, we addressed these problems by combining a dual filter extraction step, high sensitive MS, and spectra-to-spectra database searches to identify for the first time *S*. *pneumoniae* D39*-*specific proteins during bacterial meningitis in the presence of a highly complex mouse protein background (Fig 1). We therefore restricted our analysis solely to *S*. *pneumoniae* D39 derived tandem-MS spectra and created a specific library with the SpectraST search tool. This spectra-to-spectra analysis increased significantly the reliability of our identifications compared to classical *in silico*-based searches, because interfering mouse peptides/spectra were no longer misleadingly assigned as bacterial peptides.

However, due to the low number of isolated bacteria in our approach–approximately 200,000 recovered bacteria–and the presence of mouse proteins within the samples, the MS data could only be used for identifications and present/absent predictions but not for further dynamic quantification as, *e*.*g*., accomplished by the MS1 based “area under the curve” (AUC) method. Despite these limitations we have identified more than 685 proteins in the control representing proteins from pneumococci grown *in vitro* and digested on the filter and 249 from the *in vivo* CSF samples using the SpectraST search tool (Fig 2A and 2B). Most of the proteins were detected in both sample sets, and these proteins reflect as expected the most abundant proteins of pneumococci, such as ribosomal proteins, proteins involved in DNA replication, peptidoglycan synthesis-related proteins, proteins of amino acid pathways or belonging to the incomplete TCA cycle. However, some of the identified proteins were exclusively detected in the *in vivo* CSF samples from murine infections (Fig 2B). These proteins were therefore of particular interest because they mirror the immediate response of *S*. *pneumoniae* to the extrinsic host milieu. One of the identified proteins was part of the two-component signal transduction system ComDE, a key player in the competence but also virulence of *S*. *pneumoniae*. Another protein, referred to as AliB, is a component of an Ami-AliA/AliB permease complex and has also been detected in the murine CSF samples but not on the filter control of the inoculum (Fig 2). This protein is part of the ATP-binding cassette transporter and involved in the uptake of small peptides [30]. All proteomics data can be accessed via the PeptideAtlas (http://www.peptideatlas.org/).

### Role of AliB and ComDE in pneumococcal meningitis

**Fig 3:**
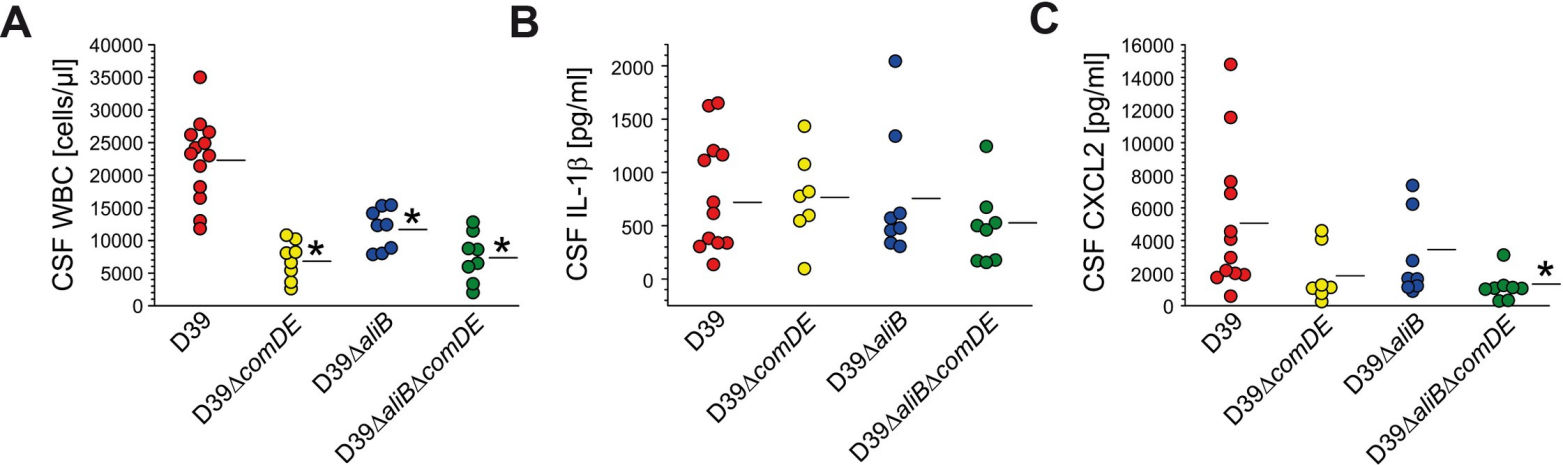
Lack of ComDE and/or AliB results in an attenuated inflammatory response to pneumococcal infection of the CSF. Pneumococcal meningitis was induced by intracisternal injection of wild type *S*. *pneumoniae* D39 (n = 13) or its isogenic ComDE-deficient, AliB-deficient- or AliB-ComDE-double-deficient mutants (each mutant n = 8). Eighteen hours later, CSF white blood cell (WBC) counts **(A)**, IL-1β **(B)**, and CXCL2 **(C)** levels were determined. **(A)** Mice infected with the single or double mutants showed significant decreases in CSF pleocytosis, as compared to D39 infected mice. **(B)** CSF IL-1β concentrations were quite similar in all mice, irrespective of whether mice were infected with D39 or its isogenic mutants. **(C)** Conversely, mice infected with mutant strains had lower CSF CXCL2 concentrations than those infected with D39, albeit only the difference between mice infected with the double-deficient mutant and the wild-type strain reached statistical significance. In negative controls (mice injected i.c. with PBS instead of *S*. *pneumoniae*; n = 8), CSF leukocyte counts were 284 ± 188 cells/μl, whereas IL-1β and CXCL2 levels were below the detection limit. Data are given as means ± SD. * P < 0.05, compared to mice infected with the D39 strain, using One-Way ANOVA and Tukey post-hoc test.

To decipher the functional role of AliB and ComDE in pneumococcal meningitis, we infected mice intracisternally with 10^5^ cfu of *S*. *pneumoniae* D39 or its isogenic ComDE-deficient, AliB-deficient- or AliB-ComDE-double-deficient mutants (S1 Fig). D39 injection caused a significant elevation of CSF leukocyte (WBC) counts (22,454 ± 6,356 cells/μl) compared to our PBS (phosphate-buffered saline) control (294 ± 188 cells/μl) at 18 h post-injection, indicating pneumococcal meningitis (Fig 3A). Mice infected with ComDE-deficient, AliB-deficient- or AliB-ComDE-double-deficient mutants showed significant decreases in CSF pleocytosis (Fig 3A).

We obtained divergent results for CSF concentrations of IL-1β and CXCL2. These cytokines are implicated in leukocyte recruitment to the CSF during experimental pneumococcal meningitis [9, 31]. Mice infected with mutant strains had lower CSF CXCL2 concentrations than those infected with the wild-type D39 strain, albeit only the difference between mice infected with the double-deficient mutant and the wild-type strain reached statistical significance. Contrarily, CSF IL-1β concentrations were quite similar in all mice, irrespective of whether mice were infected with the wild-type D39 or its isogenic mutants (Fig 3B and 3C).

**Fig 4:**
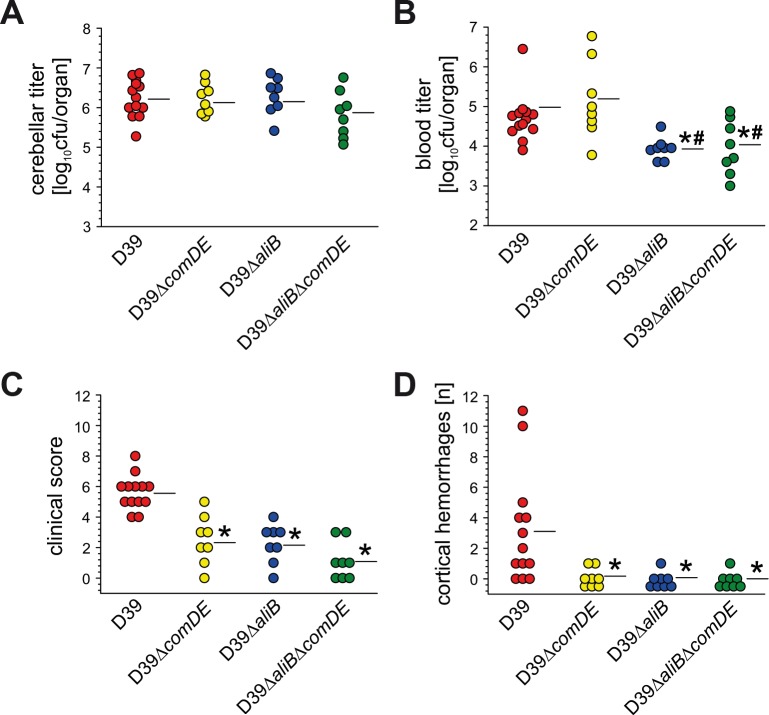
Loss of function of ComDE and/or AliB is protective in murine pneumococcal meningitis. Pneumococcal meningitis was induced by intracisternal injection of wild type *S*. *pneumoniae* D39 or its isogenic ComDE-deficient, AliB-deficient- or AliB-ComDE-double-deficient mutants. Eighteen hours later, the bacterial titres in the brain **(A)** and blood **(B)**, the clinical status **(C)**, and the number of cerebral haemorrhages **(D)** were evaluated. **(A)** The bacterial outgrowth within the CNS was comparable in all mice, irrespective of whether mice were infected with the wild-type D39 or its isogenic mutants, **(B)** whereas mice infected with AliB mutants had significantly lower bacterial concentrations in the blood. **(C)** Mice infected with ComDE-deficient, AliB-deficient- or AliB-ComDE-double-deficient mutants showed reduced illness (indicated by lower clinical score values), as compared to D39 –injected mice. **(D)** The ameliorated clinical course seems to be due to less pronounced brain pathology, as indicated by lower numbers of cerebral haemorrhages in mice infected with the mutant strains. Negative controls (mice injected i.c. with PBS instead of *S*. *pneumoniae*; n = 8) exhibited a clinical score of 0. Moreover, bacterial culturing and brain examination for the presence of haemorrhages revealed negative results. Data are given as means ± SD. * P < 0.05, compared to mice infected with the D39 strain, using One-Way ANOVA and Tukey post-hoc test.

The less pronounced CSF pleocytosis in mice infected with the mutants could also be due to lower growth rates in the brain and the blood. This might be caused by a decrease in bacterial fitness under these specific conditions. However, we found similar amounts of pneumococci in brains of all infected mice, irrespective of whether they received a mutant or the wild-type strain (Fig 4A). This is in accordance with our findings that the growth rates of pneumococcal wild-type and mutants in chemically-defined medium (CDM) and CSF were similar (S2 Fig). Strikingly, intracisternal inoculation of the mutants D39Δ*aliB* or D39Δ*aliB*Δ*comDE* was accompanied by significantly lower bacterial concentrations in the blood as compared to bacterial titers found for the wild-type D39 and mutant D39Δ*comDE* (Fig 4B).

We further measured physiological parameters like body temperature and body weight as well as spontaneous motor activity to assess murine fitness post infection. In addition, we clinically evaluated mice using an established scoring system. A clinical score of 0 indicates an uninfected and healthy mouse, and a score of 12 is attributed to terminally ill mice. Our intracisternal infection of mice with wild-type pneumococci led to a significant reduction in motor activity (4.5 ± 4.1 fields/min), body temperature (36.8 ± 0.4°C), and body weight (−11.3 ± 2.0%), which was paralleled by an increased clinical score (5.6 ± 1.1), as compared to uninfected control mice (40.0 ± 13.4 fields/min, 37.7 ± 0.3°C, + 1.6 ± 2.6%, and 0.0 ± 0.0, respectively). Mice infected with the mutant strains showed significantly higher motor activities (S3 Fig) and body temperatures as well as lower clinical score values compared to D39-infected mice (Fig 4C), and thus less clinical impairment. Loss of body weight was significantly less pronounced in mice inoculated with the double-deficient mutant than in those that had received wild-type pneumococci (S3 Fig).

Clinical impairment is—at least partly–the result of meningitis-related pathologic changes of the brain. Cortical haemorrhages are among the main pathologic findings in experimental murine pneumococcal meningitis [32]. The number of (visible) hemorrhagic spots was significantly lower in mice infected with the respective mutant strains than in mice infected with wild-type pneumococci (Fig 4D).

### Phenotypic characterization of AliB and ComDE deficient pneumococci

**Fig 5:**
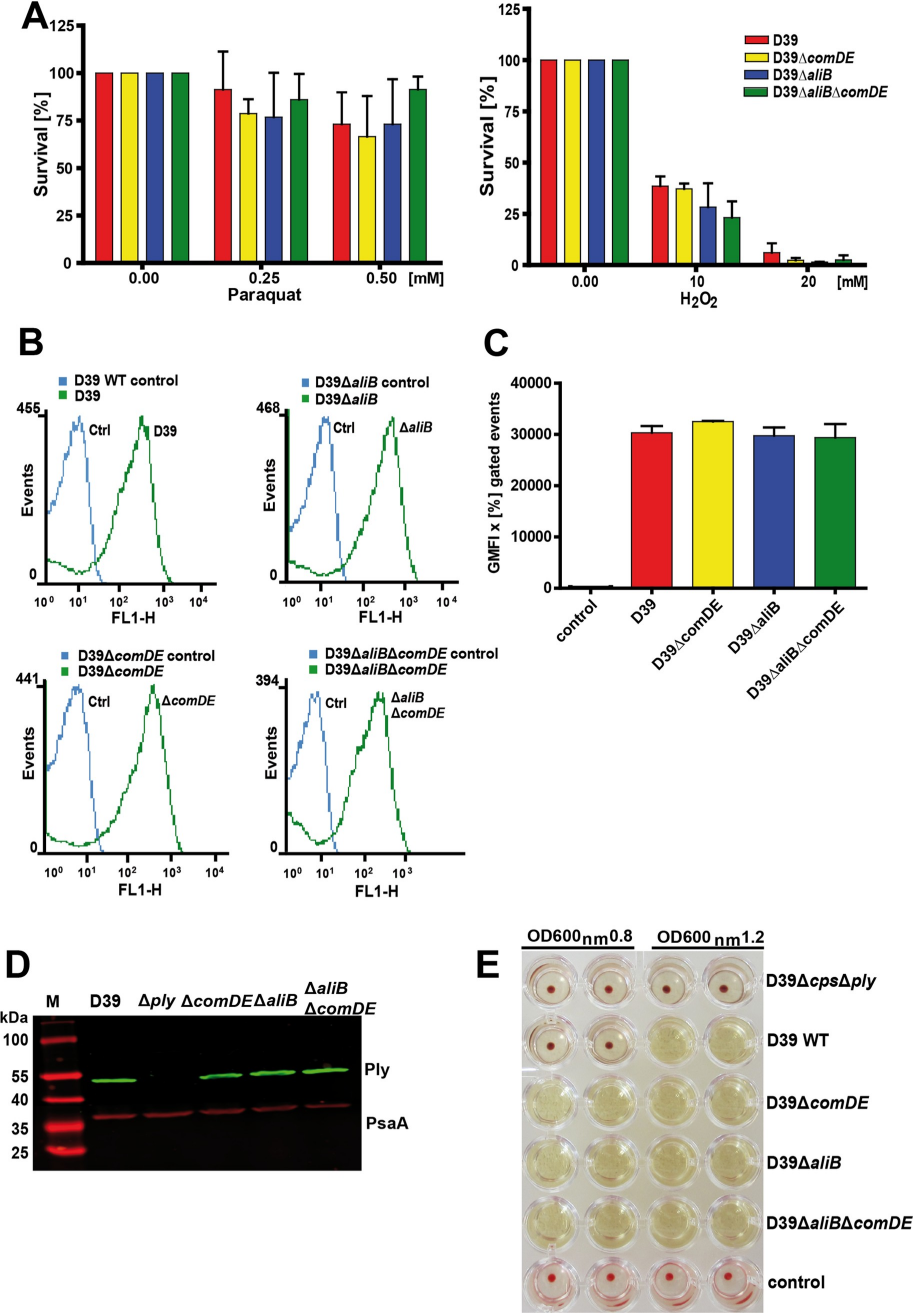
Stress resistance and virulence factor expression of ComDE and AliB-deficient pneumococci. **(A)** Stress resistance and robustness of pneumococci deficient in AliB, ComDE, or AliB and ComDE against hydrogen peroxide (H_2_O_2_) and superoxide producing compound paraquat. **(B)** and **(C)** Determination of the relative amount of pneumococcal capsular polysaccharide (CPS) on the surface of D39 and its isogenic *S*. *pneumoniae*Δ*comDE-*, Δ*aliB-* and Δ*comDE*Δ*aliB*-mutants using a flow cytometry approach. An anti-serotype 2 specific antiserum and a goat anti-rabbit IgG coupled Alexa-Fluor-488 were used to detect the CPS. A similar increase of fluorescence intensity (FL-1-H) measured for the wild-type D39 and its isogenic mutants indicated similar amounts of CPS on the surface of all tested pneumococci. Shown are flow cytometry histograms of representative experiments in **(B)** and in **(C)** the results of three independent experiments (n = 3). **(D)** The relative amount of the pneumococcal toxin pneumolysin was investigated by immunoblot analysis using a specific mouse anti-pneumolysin or PsaA (loading control) antiserum and secondary anti-mouse IgG conjugated IRDye 800CW for Ply detection and a secondary anti-mouse IgG coupled with IRDye 680RD for PsaA detection. **(E)** The haemolytic activity of pneumolysin in wild-type D39 and its isogenic mutants was compared by applying a haemolysis assay. Pneumococci were cultured in THY to OD_600_ of 0.8 or to OD_600_ of 1.2, and the supernatants were incubated for 30 min with red blood cells. The haemolytic activity is indicated lysis of the red blood cells while a red blood cell pellet was formed in absence of haemolytic activity.

The attenuation of mutants deficient in AliB or ComDE in the experimental meningitis model could be associated with dramatic phenotypic changes, lower resistance against oxidative stress, or alteration in key virulence factor expression. Because growth rates in CDM or CSF were similar, we further compared the growth of wild-type and isogenic mutants under different stress conditions. We determined survival rates of mutants deficient for AliB, ComDE or both, AliB and ComDE, in the presence of various concentrations of the oxidizing agents hydrogen peroxide (H_2_O_2_) or the superoxide generating paraquat (methyl viologen; [(C_6_H_7_N)_2_]Cl_2_). Our comparative analysis revealed similar survival rates for all mutants and the wild-type D39 suggesting that free reactive oxygen species during meningitis did not impair bacterial outgrowth in CSF (Fig 5A). In addition, we analysed the relative amount of capsular polysaccharide (CPS) by flow cytometry and the production of pneumolysin by immunoblot analysis and a haemolysis assay. The results demonstrated similar amounts of CPS and pneumolysin for the mutants and wild-type D39 (Fig 5B–5E). Thus, pneumococcal fitness and key virulence determinants were unaffected under the selected *in vitro* conditions.

### *In vitro* proteome analysis of pneumococci lacking functional AliB or ComDE

**Fig 6:**
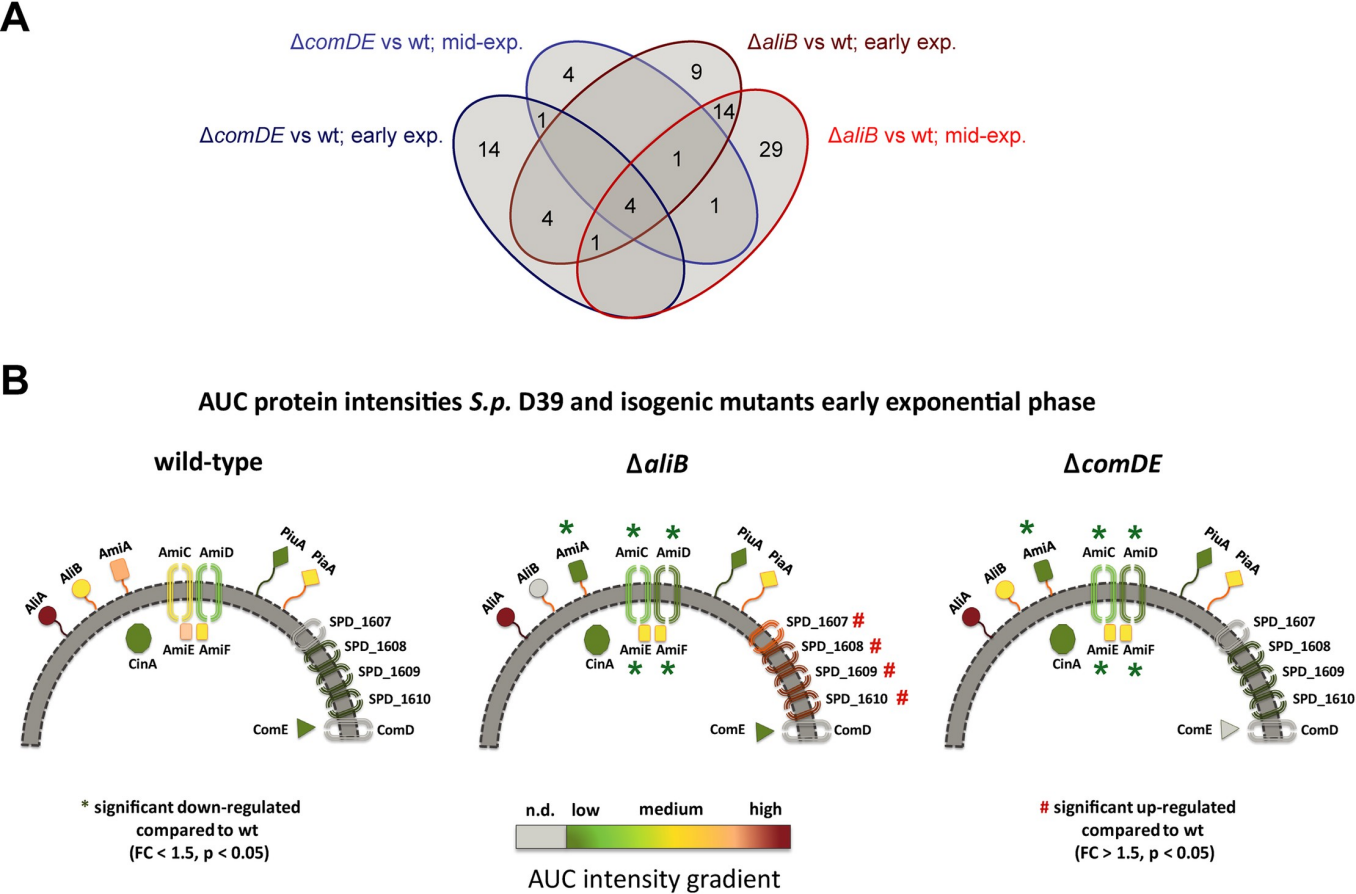
Comparison of proteins with differential abundance between *S*. *pneumoniae*Δ*comDE* or Δ*aliB* strains and the wild-type (wt) strain using a Venn diagram. **(A)** Comparison of proteins with differential abundance between Δ*comDE* or Δ*aliB* strains and the wild-type (wt) strain in early exponential (early exp.) and mid-exponential (mid-exp.) growth phase using a Venn diagram. **(B)** The schematic models show interesting proteins involved in the infection process. The intensities of the proteins were calculated from the mass spectrometry data as area under the curve (AUC). A low concentration of the protein in the sample is shown as green and a high concentration as red. Significantly regulated proteins compared to the wild-type are marked with * or #.

*In vitro* comparisons between the AliB-deficient or ComDE-deficient and isogenic wild-type pneumococcal strains were performed in the two phases of early (OD_600_ 0.12 to 0.15) and mid-exponential growth (OD_600_ 0.45 to 0.5) in a chemically-defined medium [33] resembling the nutrients available in CSF. Proteins displaying a combination of a p-value less than 0.05 and a fold change of at least 1.5 or with status present/absent (on/off) were considered as significantly regulated. Taking all the data together, a total of 1150 proteins were identified, which is roughly 70% of the theoretical proteome. Only a very small proportion of these proteins displayed significantly different levels between the different mutants and the wild-type in the different growth phases. The abundance of 24 and 11 proteins differed between ComDE-deficient pneumococci and the wild-type in early and mid-exponential phase, respectively (Fig 6A, S1 Table and S5 Table). When comparing AliB-deficient pneumococci with the wild-type, 33 proteins differed in abundance during early exponential growth and 50 proteins during mid-exponential growth phase (S1 Table). In general, all bacterial samples showed high levels of AliA, which is the substrate-binding protein of the oligopeptide ABC transporter (Fig 6A).

Beside other proteins, the Ami oligopeptide ABC transporter consisting of AmiA, AmiC AmiD, AmiE, and AmiF showed significant lower abundances in the ComDE-deficient pneumococci during early exponential growth compared to the wild-type. These proteins represent the substrate-binding protein, ATP-binding proteins and permeases encoded in a single operon (*spd*_1666, *spd*_1667-1668-1669-1670) [30, 34].

Proteins that increased in abundance included, for example, PyrF, PyrE, PyrDb, PyrB, and PyrR of the pyrimidine metabolism pathway (Spd_0608-0609-0852-1131-1133-1134) and the response regulator RR4 (Spd_1908 or PnpR). The increased abundance of the Pyr-proteins was not detected anymore during mid-exponential growth. AmiA, AmiC, AmiD, AmiE, and AmiF were still detected at slightly decreased levels during mid-exponential growth phase in ComDE-deficient pneumococci when compared to the wild-type (S1 Table). AmiF is included in the list of proteins with decreased abundance during mid-exponential phase despite their fold change is only −1.498, which did not pass the cut-off. Nevertheless, with this very small difference to the cut-off value of −1.5 also AmiF can be regarded as decreased. In contrast, AmiA seems to be switched off.

Interestingly, the oligopeptide ABC transporter proteins AmiE, AmiD, AmiC as well as AmiA but not AliA displayed decreased abundances in AliB-deficient pneumococci when compared to the wild-type during early exponential and mid-exponential growth (Fig 6B). Similar to the *com*DE mutant, the abundance of AmiA and AmiF was only significantly affected during early exponential phase in the AliB-deficient strain, but did not pass the fold change cut-off in mid-exponential phase (fold change switched off and fold change −1.27).

Relatively high increases in protein abundance (fold change 24.6 to 107.5 or switched on) were observed in AliB-deficient pneumococci for the ABC transporter permease (Spd_1607), ABC transporter ATP-binding protein (Spd_1608), ABC transporter substrate-binding protein (Spd_1609), and a hypothetical protein (Spd_1610) during early exponential as well as mid-exponential phase (S1 Table). The proteins Spd_1606–1610 are all encoded in the operon 804 (Fig 6) [35]. The MgtC/SapB family protein (Spd_1606) was not detected with our approach.

## Discussion

Pneumococci conquer various human host compartments and dissemination as well as survival requires the pathogen´s adaptation to the specific pathophysiological conditions of their environment [15]. Critical factors for pneumococcal fitness are the availability of nutrients such as carbon sources, amino acids, or metal ions [33]. In addition, pneumococci have also evolved strategies to escape the immune defence system of the host. This is undoubtedly as important as physiological fitness, because pneumococci are, depending on the stage of colonization or invasive infection, exposed to various factors of the immune system. Hence, it is essential that the repertoire of fitness and virulence factors expressed by pneumococci is adapted to the environmental cues of the host compartment. Only a tightly regulated adaptation process enables a highly efficient consumption of available nutrients, which is accompanied by *de novo* synthesis of *e*.*g*., amino acids. Furthermore, alterations of the repertoire of virulence factors provoke modulation of the host response and prevent killing by host immune factors [36].

In this proteome-based study we have recovered pneumococci from the CSF of mice and identified pneumococcal proteins that are abundant or upregulated during experimental pneumococcal meningitis. The CSF is a water-like, crystal clear fluid with very few cells (below 5 cells/μL), a low protein content (approximately 0.2% of blood total protein) as well as a lower pH, lower glucose and cholesterol concentrations, but higher chloride concentrations, as compared to the blood [37–39]. Following pneumococcal infection, substantial numbers of blood-borne leukocytes are recruited into the CSF. The CSF protein concentration increases and the protein and ion composition changes dramatically, both mainly due to the breakdown of the blood-CSF barrier. In addition, glucose levels can go down as low as zero [40, 41]. The dynamics and complexity of these CSF alterations can hardly be mimicked under *in vitro* conditions, underlining the need of *in vivo* investigations. We hypothesized that the global proteome analysis of pneumococci isolated from the CSF will identify proteinaceous factors involved in the pathophysiology of meningitis and that the results will support the understanding of molecular mechanisms of this life-threatening disease. With our experimental setup the pneumococcal oligopeptide transporter AliB and the two-component regulatory system ComDE were exclusively detected in the *in vivo* CSF compartment. However, this does not exclude that these proteins may not be produced under different *in vivo* conditions. The contribution of AliB and ComDE to pneumococcal colonization, but not invasive disease, has been demonstrated earlier [42, 43]. However, their role in pneumococcal meningitis had not been studied so far. Thus, we explored the influence of the oligopeptide transporter AliB and the two-component regulatory system ComDE on pneumococcal meningitis. Our results clearly proved their critical role in the development of severe pneumococcal meningitis. The significant attenuation of pneumococcal mutants in the experimental meningitis model did not rely on growth defects or diminished expression of crucial virulence factors such as the capsule or cytotoxin pneumolysin. Because of its important role in pneumococcal meningitis [9, 44], a reduction in pneumolysin production would have been an explanation for the significantly reduced clinical scores and cortical haemorrhages in the presence of similar cerebellar bacterial titres. In addition, the robustness of pneumococci lacking AliB or ComDE expression against oxidative stress was not affected, suggesting that the induced reactive oxygen species during meningitis are not the limiting factors. Strikingly, the *comDE*-mutant showed similar CFU in the blood compared to the wild-type, whereas the *aliB*-mutant had significantly lower blood titres, pointing to the inability of the *aliB*-mutant to transmigrate form the CSF into the blood in our model. Interestingly, non-encapsulated pneumococcal strains produce AliB-like proteins, named AliC and AliD. These strains are associated with colonization and non-invasive diseases including non-bacteremic pneumonia [45, 46]. Consistent with these findings mutants with loss of function of AliC or AliD did not cause pneumococcal meningitis [47].

A substantial limitation of *in vivo* transcriptomics and our *in vivo* proteomics approach was the low number of pneumococci recovered from the distinct *in vivo* host compartments. Other *in vivo* proteomics studies faced identical problems, when investigating pneumococcal proteins in the presence of host proteins, and identified therefore a very small number of bacterial proteins. The authors clarified that *in vivo* proteome studies of pathogenic pneumococci will only be feasible with new and specialized techniques [48]. In a former study, when analysing the proteome of *Staphylococcus aureus*, we had the same experience [25]. In this study we therefore developed a new filter enrichment and spectral library evaluation strategy to cope with the small amounts of bacteria. With this method, we were able to extract a maximum of 50,000 bacteria from the CSF of a single mouse. Because this is still a relatively low number of bacteria, we have tried to increase the amount of infected CSF to be filtered. However, this ended up with an excess of host proteins, which were increasingly identified in MS.

In spite of the presence of eukaryotic proteins and low numbers of bacterial proteins, the dual filter extraction step and the generation of a highly specific pneumococcal SpectraST library enabled us to identify 249 proteins even in the presence of a large excess of host proteins under *in vivo* conditions. To put this in relation, in a similar approach 578 proteins were identified from *Staphylococcus aureus* recovered from the upper respiratory tract of intranasally infected mice [25]. However, while we have identified in total only 685 pneumococcal proteins by on-filter digestion from *in vitro* experiments, Michalik *et al*. identified in total 1,719 proteins over all samples after sorting of 5 x 10^6^ *S*. *aureus* cells by on-filter digestion. These differences are probably due to the higher number of recovered bacteria, a higher robustness of *S*. *aureus* compared to the autolytic pneumococcus and a higher efficiency of the tryptic digest with *S*. *aureus*.

A prerequisite for our robust *in vivo* proteomics approach was the generation of a SpectraST library, which contains high number and high quality tandem-MS reference spectra for the organisms of interest. Our comprehensive pneumococcal SpectraST library contained 49,083 tandem-MS spectra reflecting 1,165 protein identifications. Similar to our study a *S*. *aureus* SpectraST library from a comprehensive set of peptide data was used for the identification of *S*. *aureus* proteins [49]. Those SpectraST libraries and the appropriate PeptideAtlas entries are valuable proteome repositories facilitating in mixed host-pathogen samples or in contaminated samples targeted proteome analysis and if possible, proteome quantification [25, 49].

We were intrigued to identify AliB and ComDE as upregulated bacterial factors when pneumococci disseminate in the CSF. Several studies have already addressed pneumococcal adaptation and its *in vivo* host-compartment specific gene expression by examination of the transcription pattern. In addition to genome-wide *in vivo* transcriptomic analyses other sophisticated molecular techniques have been employed to identify key pneumococcal players under *in vivo* conditions. One of these studies identified upregulated genes in the nasopharynx, lungs or blood such as e.g., *aliA*, *comB* and other competence genes [24]. This study further showed that immunization with AliA protects against pneumococcal infections. A follow-up microarray analysis identified genes differentially expressed in the nasopharynx and blood such as the genes encoding the superoxide dismutase, the serine protease PrtA, proteins part of transport systems as well as ComE [22]. Using an experimental rabbit meningitis model and the microarray-based GAF (genomic array footprinting) technology genes were identified that were attenuated during meningitis. Strikingly, this approach did not identify competence genes or genes encoding proteins of the Ami system including AliA and AliB [23]. More recently, the transcriptome of *S*. *pneumoniae* D39 has been analysed under 22 different infection-relevant, but not *in vivo* conditions including growth in CSF-mimicking conditions. Intriguingly, *comDE* is significantly upregulated in CSF mimicking conditions and cell culture infection experiments with A549 cells (https://veeninglab.com/pneumoexpress-app/index.php). In contrast genes of the Ami-AliA-AliB system have not been identified to be upregulated in CSF-mimicking conditions, suggesting that the *in vivo* host environment plays a fundamental role in modulating gene expression [50].

ComDE is one of the 13 two-component regulatory systems (TCS) of pneumococci (TCS12) and is a master regulator of competence. However, ComDE is further involved in antibiotic resistance and regulation of virulence factors[51–54]. Competence is auto-controlled via the *comCDE* operon, where *comC* encodes the proactivator that is exported by the ComAB machinery [55]. The mature form of ComC, known as competence stimulating peptide, activates the histidine kinase ComD, which subsequently leads to activation of the response regulator ComE [52]. Importantly, ComE regulates early competence genes like *comX* and *comW*, which in turn activate the expression of late competence genes including those encoding the peptidoglycan hydrolases CbpD and LytA. To avoid committing suicide, competent pneumococci protect themselves against CbpD by expressing the immunity protein ComM, which is encoded by an early competence gene [56]. The link between competence and virulence has been indicated by the impairment of pneumococci deficient in ComD or ComX to cause pneumonia and bacteraemia in mice [18, 54, 57]. Here, we show in addition that regulation of competence genes is also crucial for pneumococcal meningitis, probably by the mode of action of the alternative sigma factor ComX, which regulates genes associated with cell wall turnover and virulence [54, 58].

Induction of the competent state in *S*. *pneumoniae* leads to the expression of several proteins that alleviate stress, e.g. the heat shock proteins ClpL, GrpE, DnaK, DnaJ, and HtrA that function to disaggregate, refold, and/or degrade misfolded proteins [59, 60]. Interestingly, the CiaR and LiaR regulons, which are activated in response to cell envelope stress, are both upregulated in competent pneumococci. Presumably, stress proteins are expressed to alleviate the temporary stress imposed during the competent state. However, since some types of external stress can induce competence, it is also possible to regard competence as a stress response mechanism itself [59, 61–64]. Hence, the upregulation of ComDE and competence observed in pneumococci during meningitis might be a stress response mounted by the bacteria to defend themselves against attacks from the host immune system.

AliB is a lipoprotein and substrate-binding protein (SBP) of the Ami system, the only oligopeptide ABC transporter system identified in pneumococci. Other oligopeptide-SBPs of the Ami system are the lipoproteins AmiA and AliA, sharing approximately 60% identity to AliB [30]. A triple mutant devoid of all three oligopeptide-SBPs is impaired in oligopeptide uptake and interestingly, the lack of AliB reduced competence [30, 34]. However, it is important to mention here that the Ami system is not directly involved in the response to the competence stimulating peptide CSP encoded by ComC [65]. The Ami system further consists of the ATP-binding proteins AmiE and AmiF and the permeases AmiC and AmiD and loss of function in AmiDEF inhibited uptake of all tested oligopeptides [30]. Other studies identified an *aliB*-like ORF in the capsule region of non-encapsulated pneumococci [66], which may compensate the deficiency in the three SBPs of the Ami system. While *amiABCDEF* are part of a polycistronic operon, AliA and AliB are located in other genomic regions and are independently regulated from each other and AmiA.

Growth studies and *in vivo* infections indicated that loss of function of AmiA and AliA can be compensated *in vitro* and *in vivo* by AliB. Despite that all three oligopeptide-SBPs are required for successful colonization of the nasopharynx, the deficiency in AliB had the slightest impact on colonization, while AliA or AmiA affected colonization dramatically [30, 42]. This suggested differences in their substrate specificity. Indeed, the *aliB*-mutant was unable to grow on Arg-Pro-Pro and Arg-Pro-Pro-Gly-Phe as oligopeptides, while pneumococci lacking AliA and AliB could use only Leu-Leu-Leu oligopeptides. A substrate-specificity for AliB has been suggested for peptides Arg-Pro-Pro and Leu-Arg-Arg-Ala-Ser-Leu-Gly, respectively [30]. In a recent study Ami-AliA/AliB ligands have been identified. AmiA binds peptide AKTIKITQTR, AliA peptide FNEMQPIVDRQ and AliB peptide AIQSEKARKHN. Interestingly these peptides are part of 50S or 30S ribosomal proteins found in species of γ-proteobacteria, which are commensals of the upper respiratory tract [47].

Despite the functional redundancy between AmiA, AliA, and AliB, we could not observe a compensation of loss of function of AliB in our experimental meningitis infection model. The *aliB*-mutant is attenuated as indicated by significantly decreased bacterial titres in the blood, lower clinical score values, and less pronounced brain pathology. This suggests that under the given conditions in the meningitis model, AmiA and AliA cannot fully compensate for the loss of AliB. The absence of AliB leads to uptake of a reduced spectrum of oligopeptides, which presumably results in the deficiency of essential amino acids. This in turn leads to lower fitness and lower virulence during infection of the subarachnoid space. This is in contrast to a colonization model, in which AliB had only a minor effect, while the impact was more pronounced for AmiA and AliA [42]. However, we are not aware of any study that has assessed the individual impact of AmiA or AliA on pneumococcal meningitis. Importantly, our *in vitro* proteome analysis of the mutant deficient in AliB revealed that the amount of AliA is high in the wild-type and the *aliB*- or *comDE*-mutant as well. In contrast, the protein amount of AmiA is significantly decreased in the early and mid-exponential growth phase of *aliB*-mutants. Surprisingly, AmiA and its cognate permease as well as ATPase are also significantly reduced in the *comDE*-mutant of D39. The reason for this coincidence is not clear, but may contribute to the attenuation of our mutants.

AmiA is also affected by loss of function of AliB. This probably further reduces the spectrum of oligopeptides that can be taken up by pneumococci as has been shown earlier [30]. Because pneumococci are auxotrophic for several amino acids [33, 67] this has consequences for nutrition and protein synthesis. Under conditions where the amino acid pool is unbalanced mischarging of tRNAs takes place [68] and premature termination of protein synthesis will happen. Hence, in an *aliB*-mutant, where the expression of AmiA is strongly reduced as well, mischarging of tRNAs and premature termination will lead to the production of truncated and misfolded proteins due to declined uptake of amino acid [30]. This situation is most likely stressful for pneumococci as indicated by an upregulation of ClpL (ATP-dependent Clp protease; Spd_0308) in the *aliB*- and *comDE*-mutant as well. In contrast, another protease, the extracytoplasmic protease HtrA, which represses competence by digestion of CSP, was not significantly altered.

While the Ami system is important for uptake of peptides, other ABC transporters, as for instance iron transporters, are crucial for ion homeostasis like iron transporters. Pneumococci acquire iron via PiaABC and PiuABC, however, the proteome analysis indicated no changes in abundance in mutants deficient in AliB or ComDE. Similar, other transporters were not affected with the exception of the PTS system transporter protein SPD_0661 (2-fold) and a potential ABC iron-transporter system encoded by the operon 804. This operon encodes the proteins SPD_1606 to SPD_1610 with the lipoprotein SPD_1609 as SBP of the system [35]. Proteins encoded by this gene cluster were more than 20-fold upregulated in the *aliB*- but not *comDE*-mutant, suggesting a compensatory effect.

## Material and methods

### Ethics statement

This study was carried out in strict accordance with the recommendations in the Guide for the Care and Use of Laboratory Animals (National Research Council, USA) and with the German Animal Protection Act. The study protocol was approved by the Committee on the Ethics of Animal Experiments of the Government of Upper Bavaria (Permit Numbers: 55.2-1-54-2531-31-09 and 55.2-1-54-2531-143-12). All efforts were made to minimize suffering, ensure the highest ethical standards, and to adhere to the 3R principle (reduction, refinement and replacement).

### Bacterial strains and growth conditions

Bacterial strains and recombinant plasmids used in this study are listed in S2 Table. *Streptococcus pneumoniae* was cultivated in Todd-Hewitt broth (Roth) supplemented with 0.5% yeast extract (THY) or in chemically-defined medium (RPMI_modi_ = CDM) [33] without agitation at 37°C. Serotype 2 strain D39 was chosen for the *in vivo* study because the disease course and pathology associated with this serotype has been extensively studied in rodent models (in which the bacteria are directly injected into the cerebrospinal fluid or the brain), thus allowing broad comparison of results [69]. Genetically modified pneumococci were selected on Columbia blood agar plates (Oxoid) supplemented with 5% defibrinated sheep blood, and antibiotics were added as appropriate including kanamycin (Km 300 μg/ml) and chloramphenicol (Cm 2 μg/ml). Recombinant *E*. *coli* were grown in Luria–Bertani (LB) medium at 30°C under agitation (110 rpm) or on LB agar plates. The following antibiotics were added for different recombinant *E*. *coli*: Km 50 μg/ml, Cm 20 μg/ml).

### Growth and survival of *S*. *pneumoniae* under oxidative conditions

Pneumococcal wild-type D39 and isogenic mutants D39Δ*aliB*, D39Δ*comDE* or D39Δ*aliB*Δ*comDE* were cultured in complex THY medium at 37°C up to mid-exponential growth phase (OD_600nm_ 0.5–0.6). Bacterial cultures were then splitted and hydrogen peroxide (final concentration 10 mM or 20 mM) or paraquat (final concentration 0.25 mM or 0.5 mM) was added, and samples were incubated at 37°C for 30 min [70]. Untreated pneumococcal cultures were used as control. Serial dilutions were then plated on blood agar plates and incubated overnight at 37°C and 5% CO_2_. Survival rates of mutants were determined by counting the cfu and comparing them to the cfu of untreated bacteria.

### Protein extraction and enzymatic digestion of samples from *in vitro* experiments

To prepare a qualitative pneumococcal SpectraST library we cultured D39 and TIGR4 as well as pneumococcal mutants (Δ*cps*; Δ*rex*) pneumococci under various conditions including higher temperature (40°C) and various stress conditions (hydrogen peroxide, sodium hypochloride, and paraquat). Cell disruption of bacteria was performed using a bead mill, a freezing and thawing procedure, or by direct tryptic digestion after transfer of the bacteria on a filter membrane. For the first and second method we determined protein concentrations in the resulting cell lysates and performed a tryptic digestion with 4 μg of protein per sample. The filter membrane digestion was performed with a cell count of approximately 10^7^ bacteria to reach sufficient protein quantities. Afterwards, the crude peptide mix from all cell disruption methods was purified and desalted with C_18_ ZipTip material (Merck Millipore, Merck KGaA, Darmstadt, Germany).

### *In vivo* infection of mice and bacterial isolation via dual filter extraction

We used a well-characterized mouse model of pneumococcal meningitis [31, 71]. Briefly, 8–12 weeks old male C57BL/6n mice were weighed and their body temperature was recorded. Their spontaneous motor activity was determined using an open field test device. Then, mice were clinically examined and scored. Clinical scoring comprised [i] a beam balancing test, [ii] a postural reflex test, [iii] the presence of piloerection, seizures or reduced vigilance. In healthy animals, the score is 0 points; twelve points are attributed to terminally ill animals that have to be euthanized and excluded from the experiment. After clinical evaluation, all mice received analgesic therapy with buprenorphine. One hour later, we induced meningitis by intracisternal injection of 10 μl of *S*. *pneumoniae* suspension containing 10^7^ colony forming units (cfu) per ml under short term anaesthesia with isoflurane. Mice were infected with the following pneumococcal strains: D39, D39Δ*aliB*, D39Δ*comDE*, and D39Δ*aliB*Δ*comDE*. For virulence experiments, mice were randomly allocated to the four different groups by drawing cards before infection. Animals were allowed to wake up, and food and water were supplied *ad libitum*. At 18 hours after infection, mice were clinically examined and then anaesthetized with ketamine/xylazine. A catheter was placed into the cisterna magna. CSF samples were withdrawn either to recover bacteria for proteomics analysis or to determine CSF leukocyte counts as well as CSF IL-1β and CXCL2 concentrations for virulence analyses of mutant strains. After intraperitoneal pentobarbital injection, chests were opened, and blood samples were taken by transcardial puncture. Thereafter, mice were perfused with 15 ml ice-cold PBS containing 10 U/ml heparin. The brains were removed and assessed for the presence of visible cortical haemorrhages. The cerebella were dissected and homogenized in sterile saline. Blood samples and cerebellar homogenates were diluted serially in sterile saline, plated on blood agar plates, and cultured for 24 h at 37°C with 5% CO_2_. All mentioned outcome parameters were assessed by an investigator blinded to group allocation.

### Measurement of CSF leukocyte counts, CSF IL-1β, and CXCL2 concentrations

Two μl of CSF were diluted with 18 μl Turk’s solution and used for leukocyte counting in a Fuchs-Rosenthal chamber. The remaining CSF was diluted 1:20 in PBS. Fifty μl of each sample were used to quantify IL-1β and CXCL2 concentrations by ELISA (R&D Systems, Wiesbaden-Nordenstadt, Germany).

### Bacterial sampling from infected mice

To isolate and enrich pneumococci from the CSF of infected mice, we developed a two-step centrifugation procedure across a microfilter that allowed fast and reliable separation of bacteria and host cells from CSF samples of mice subjected to bacterial meningitis. Bacterial inoculum suspensions (used for the induction of meningitis) as well as CSF samples (mean volume 9.8 ± 3.9 μl) were diluted 1:40 in PBS containing 0.1% bovine serum albumin (BSA). The solutions were passed through Whatman Puradisc syringe filters (pore size 1.6 μm; pre-coated with BSA) in order to remove host cells. Ten μl of each filtrate were then used to enumerate bacteria by plating and culturing. The remaining filtrates were centrifuged through filter units with Durapore PVDF membranes (pore size 0.22 μm; pre-coated with BSA) in order to recover the extracellular pathogens. The bacteria trapped by the 0.22 μm filters were immediately stored at −80°C until further processing. In pilot experiments, CSF either taken from single mice or pooled from groups of 4 and 8 mice were subjected to bacterial counting and proteomics analysis in order to identify the sample size that yielded the highest detection rate of bacterial proteins. The mean numbers of bacteria recovered from CSF samples taken from one, 4, and 8 mice accounted to 49,100 ± 27,200 cfu, 317,600 ± 50,500 cfu, and 350,500 ± 78,500 cfu, respectively. Subsequent proteome analyses revealed that the bacterial numbers obtained from a single mouse were too low to allow protein detection, whereas the results were similar in samples pooled from 4 or 8 mice with approximately 200 detectable proteins in each group.

### *In vitro* growth of Δ*comDE* and Δ*aliB* mutants and their corresponding wild-type strain for harvest of samples intended for proteomics growth-phase-specific strain comparisons

Pneumococci were cultivated sequentially on blood agar plates, first for 8 h and afterwards on fresh plates for another 10 h. RPMI1640 medium was inoculated to an OD_600nm_ of 0.06 using bacterial material from the blood agar plates. Bacteria were grown at 37°C without shaking. Samples were harvested at early exponential growth phase (OD_600_ 0.12 to 0.15) and at mid-exponential growth phase (OD_600_ 0.45 to 0.5) by centrifugation. Bacterial cells were washed with PBS and stored at −80°C. Bacterial cell disruption and preparation of protein extracts were performed as described above for samples used for building the SpectraST library.

### Protein extraction and enzymatic digest from dual filter extraction *in vivo* experiments

Bacteria derived from CSF samples were submitted to a direct on-membrane digestion to avoid any loss of material. Each filter (Ultrafree-MC, GV 0.22 μm, Merck Milipore, Germany) was soaked in 60 μl 20 mM ammonium bicarbonate containing 13 ng trypsin and incubated overnight at 37°C. Resulting peptides were centrifuged through the filter for 5 min at 15,700 g and the digestion reaction was stopped by adding trifluoroacetic acid to a final concentration of 0.1%. Afterwards the peptide purification and desalting was achieved with C_18_ ZipTip columns.

### Mass spectrometry analysis

Peptides were dried using a vacuum centrifuge concentrator and afterwards dissolved in LC buffer A (2% acetonitrile, 0.1% acetic acid in HPLC-grade water) for the measurement. LC-MS/MS analyses of pneumococcal samples from cultivation experiments were performed by peptide separation on a Thermo Scientific Easy nLC II using a NS-MP-10 Biosphere C18 with 100 μm inner diameter and 20 mm length (Nano Separations, Netherlands) as pre-column and an Acclaim PepMap 100 with an inner diameter of 75 μm and a length of 15 cm packed with C18 material (Thermo Scientific, USA) as analytical column. Separation of peptides was achieved by a non-linear gradient starting at 2% LC buffer B (0.1% acetic acid in acetonitrile) and 98% LC buffer A with an increasing LC buffer B proportion to 5% in the first minute, to 25% until minute 60, to 40% until minute 70, and finally to 100% until minute 78. Elution was performed at a flow rate of 0.3 μl/min, and following mass analysis was performed with a LTQ Orbitrap Velos (Thermo Electron Corporation, Germany) equipped with a nano-ESI source using a Picotip Emmitter (New Objective, USA). Ionization was set to positive mode, a data-dependent acquisition Top 20 method was applied with full scans covering the m/z range of 300–1,700 at a resolution of 30,000 with a target value of 1E6. Only charge states +2 and +3 ions were subjected to fragmentation by CID with an isolation width of 2 Da and at normalized collision energy of 35%. Targeted ions were afterwards excluded from fragmentation for 60 s using a dynamic exclusion list.

LC separation of bacterial samples from on-filter digestions was conducted with a nanoAcquity UPLC (Waters Corporation, USA) using a 2G-V/M trap Symmetry C18 pre-column of 20 mm length and 180 μm inner diameter (Waters Corporation, USA) and a nanoAcquity BEH130 C18 analytical column of 10 cm length and 100 μm inner diameter (Waters Corporation, USA). Peptide separation was achieved by a non-linear gradient starting at 1% LC buffer B and 99% LC buffer A with an increasing LC buffer B proportion to 5% until minute 2, to 25% until minute 65, to 60% until minute 90, and finally to 100% until minute 91. Elution was performed at a flow rate of 0.4 μl/min, and subsequent mass analysis was performed as described above.

All raw-files were submitted to a sequence database (NCBI 2014, 1914 proteins, 145357 peptides) search using the Sorcerer platform and trans-proteomic pipeline (TPP) [72]. No static modifications were set, the peptide mass tolerance was set to ± 20 ppm, and a maximum of two missed cleavages was allowed. For SpectraST library inclusion a minimum probability of 0.95 was required.

### Mass spectrometric analysis of *in vitro* cultivation samples of Δ*comDE* and Δ*aliB* mutants and their corresponding wild type strain

Samples of *in vitro* cultivated *S*. *pneumoniae* wild-type and Δ*comDE* and Δ*aliB* mutants were analysed using an LC-MS/MS system of an UltiMate 3000RSLC (Thermo-Fisher Scientific, Idstein, Germany) and a Q Exactive Orbitrap-MS (Thermo-Fisher Scientific Inc.) in data-dependent mode. Each sample consisted of 2 μg of purified tryptic peptides. Detailed settings for LC-MS/MS are listed in S4 Table.

### Identification of peptides via spectral library from *in vivo* experiments

To identify proteins in the CSF-derived bacterial samples we submitted the raw-files to a SpectraST search tool from the Trans-Proteomics Pipeline using the described library (http://tools.proteomecenter.org/wiki/index.php?title=Software:TPP). For the selection of high quality spectra matches the result files were filtered by dot score (≥ 0.6 required) and m/z difference of ± 0.01 Da at maximum.

### Analysis of MS data from samples of Δ*comDE* and Δ*aliB* mutants and their corresponding wild type strain in different growth phases

A set of 24 MS raw data files (3 strains, 2 growth phases, 4 biological replicates per condition) was analysed using MaxQuant software package [73] with parameter settings for label-free quantitation. Trypsin/P (specific, i.e. full-tryptic) and up to 1 missed cleavage were set for digestion. Oxidation of methionine was allowed as variable modification. MS data were searched using a database of 1986 pneumococcal protein sequences (NCBI, D39, NCTC 7466), to which reversed entries and contaminant sequences were added by the MaxQuant software during analysis. Protein LFQ intensities were built and exported to Excel. After removal of contaminant, reversed, and protein group identifications, the remaining data were imported to the GeneData Analyst (GeneData AG, Basel, Switzerland) software package where log_10_-transformation and a further median normalization were conducted. Non-logarithmized values were subjected to growth phase-specific, group-wise statistical comparison between the strains using 2-group t-test under the restriction of at least 75% valid values per group (i.e. 3 of 4 replicates must have given a quantitative value). Finally, p-values and normalized intensities were exported back to Excel for calculation of mean intensity values from all 4 replicates per condition and for fold change calculation from these mean values. Proteins with p-values of 0.05 or less and absolute fold change values of at least 1.5 were regarded as regulated.

### Visualization of detected proteins by Voroni Treemap

According to their function, SEED [74] *S*. *pneumoniae* D39 gene ontology classifies genes/proteins in an acyclic multi-hierarchical tree-graph. Voronoi Treemaps [75] were used for a graphical planar representation of this tree-like structure. Our Voronoi treemap-based layout of SEED ontology intuitively visualizes the proteome coverage of general cell functions by our *in vitro* and *in vivo* proteomics based approaches.

### Molecular biology techniques

Isolation of plasmid DNA was performed as recommended by the supplier of the DNA isolation kit (Promega). PCR reactions were carried out with Taq polymerase or Pfu polymerase (New England Biolabs, Germany). Genomic DNA isolation from pneumococci was performed by a fast preparation method. Briefly, pneumococci were cultivated in THY medium to OD_600nm_ of 0.3 to 0.4. Samples of 200 μl were centrifuged at 10,000 g for 2 min. The bacterial sediment was resuspended in 100 μl PBS and centrifuged at 10,000 g for 4 min. Supernatants were carefully removed, and pneumococci were resuspended in 50 μl sterile water and incubated for 8 min at 96°C. After cooling at room temperature another centrifugation at 10000 g for 2 min was performed. Samples were then frozen at −20°C for 30 min. Two μl were used as DNA template for a PCR. Primers used in these studies were synthesized by Eurofins MWG Operon and are listed in S3 Table. Recombinant plasmids were transformed into competent *E*. *coli* DH5α treated with CaCl_2_.

### Generation of *aliB-* and *comDE*-mutants

The deletion insertion mutagenesis of *aliB* has been described previously [30]. A D39 strain harbouring the *aliB* mutation by insertion of a chloramphenicol cassette in the 5'-region of *aliB* was kindly provided by C. van der Gast, Nijmengen, Netherland. *AliB* specific primers (aliB_1276, aliB_1277) listed in S3 Table were used for amplification of the mutated *aliB* gene with Pfu polymerase. A 3.0 kb fragment was obtained and cloned into vector pSP72 digested with *Pvu*II. Recombinant *E*. *coli* DH5 harbouring the corresponding recombinant plasmid were selected on LB medium supplemented with chloramphenicol. The isolated recombinant plasmid (pSP1037) was transformed into our D39 strain by adding competence stimulating peptide-1 at a final concentration of 0.04 μg as described earlier [70], and mutants were selected on blood agar plates supplemented with chloramphenicol. Gene replacement by chromosomal integration was verified by analytical PCR using total DNA from the pneumococcal mutant and primer pair aliB_1276/aliB_1277 (S1A and S1B Fig).

For mutagenesis of *comDE* a designed PCR product containing an *aphA3* gene cassette instead of *comDE* genes was kindly provided by Reinhold Brückner, University of Kaiserslautern. The construct was generated by overlap PCR. PCR reactions were carried out to amplify upstream and downstream region of *comDE* with primers containing additional sequences of a kanamycin gene. Primer combination of ComC_fwd/comD_kan_rev2 were used to amplify 837 bp fragment upstream of *comDE* and primer combination ComE_kan_fwd2/comE_rev to amplify 831 bp downstream of *comE*. Total DNA from strain R6 as template was added. The *aphA3* cassette (0.9 kb) encoding kanamycin resistance was amplified with primer pair ComD_kan_fwd2/ComE_kan_rev2 with complementary sequence of *comD* or *comE* and RK4 DNA (*ccnD*::*aphA3*) as template [76]. The resulting PCR fragments were separated and purified from agarose gels with gel DNA recovery kit (Zymo Research). All three DNA fragments were used in an overlap PCR reaction with primer combination ComC_fwd/ComE_rev for the generation of the 2,657 bp mutant construct. Reamplification was carried out with primer pair comDE_1224/comDE_1225 and mutant construct as template to amplify a 2.1 kb fragment (*comDE*::*aphA3*), which was transformed into D39 and D39Δ*aliB* for the generation of *comDE* deletion mutants. Recombinant clones harbouring the *comDE* deletion were selected on blood agar containing kanamycin (300 μg/ml) and replacement was verified by analytical PCR using the primer combination comDE_1224/comDE_1225 (S1 Fig). The deficiency in AliB expression was also confirmed by immunoblot analysis with anti-AliB serum (S1 Fig).

### Heterologous expression of AliB, protein purification, and generation of polyclonal anti-AliB and anti-pneumolysin antibodies for immunoblotting

*The aliB* gene (SPD_1357) was amplified with primers aliB_1288/aliB_1277 (nucleotide position 76–1959). Primers contained the *Nhe*I and *Sac*I site to clone the digested PCR product into similarly digested vector pTP1 [70]. This generates an N-terminal His_6_-tag fusion to AliB. The resulting plasmid pET1033 was transformed into *E*. *coli* BL21(DE3) for heterologous protein expression of AliB. Recombinant clones were cultivated in LB medium with kanamycin at 30°C until reaching an OD_600nm_ of 0.6, and expression was induced with 1 mM IPTG (isopropyl-b-D-1-thiogalactopyranoside) for 3 h. Bacteria were lysed by sonification (3 times each 30 sec at 75%), and cytoplasmic protein extract was received after centrifugation at 16,000 x g for 20 min at 4°C. Protein extract was loaded on His Trap HP Ni-NTA column (1 ml; GE Healthcare), and purification of His_6_-AliB was performed with increasing imidazole concentration (linear range between 0–500 mM) using an Äktapurifier liquid chromatography system (GE Healthcare) according to the instructions of the supplier. The His_6_-tag was removed by TEV protease cleavage overnight in 100 mM NaCl, 50 mM NaH_2_PO_4_, pH 7.4 at 4°C as described [70], and recombinant purified rAliB was collected from the flow through by a second affinity chromatography. The purity of rAliB was analysed by SDS-PAGE and staining of the gel with Coomassie brilliant blue, and protein concentration was determined with Bradford reagent according to the manufacturer’s description (BioRad).

Purified His_6_-tagged AliB (rAliB) and purified strep-tagged pneumolysin (rPLY) were used to raise polyclonal antibodies in 6–8 weeks old female CD-1 mice by a routine immunization protocol. Briefly, 20 μg of purified rAliB or rPLY in 100 μl PBS buffer was intraperitoneally injected into the mice with an equal volume of incomplete Freund’s adjuvant (Sigma Aldrich). After two weeks same amount was injected for boosting. This was repeated one more time. After 6 weeks mice were sacrificed and serum was collected. Specificity of polyclonal anti-AliB or anti-pneumolysin antibodies was tested in immune blots. Thus, pneumococcal crude extract or purified rAliB was separated by SDS-PAGE and transferred onto nitrocellulose by semidry blotting (BIORAD). Membrane was blocked with 5% skim milk in TBS-Tween buffer (0.2 M NaCl, 50 mM TRIS, 0.5% Tween20, pH7.4). Alkaline-phosphatase goat anti mouse IgG (1:5000) (Abcam) was used as secondary antibody for the detection anti-AliB serum (1:1000) or anti-Ply (1:500). Signals were visualized with NBT/BCIP color development substrate for alkaline phosphatase activity. Detection of DacB (l,d-carboxypeptidase) or ArcB (ornithine carbamoyltransferase) was conducted with specific mouse anti-DacB or anti-ArcB polyclonal serum [33, 77] as loading control.

### Determination of the capsular polysaccharide amount by flow cytometry

Flow cytometry was applied to investigate the amount of capsular polysaccharide of *S*. *pneumoniae* D39 serotype 2. In principle, the flow cytometric analysis was carried out as described recently [70]. Briefly, pneumococci cultured in liquid media (CDM) were harvested, and 1 x 10^8^ bacteria were incubated with an anti-serotype 2 specific antiserum (Statens Serum Institute, Denmark) (1:500 dilution in PBS) for 30 min at 4°C. Samples were then washed twice with PBS/0.5% FCS and stained with secondary goat anti-rabbit IgG coupled Alexa-Fluor-488 (Abcam). After 30 min incubation at 4°C bacteria were washed twice with PBS/0.5% and then fixed with 2% formaldehyde. Flow cytometry was conducted with a FACSCalibur™ (BD Biosciences, Heidelberg, Germany), and the CellQuestPro Software 6.0. (BD Biosciences) was used for data acquisition while analysis of the data was performed with the software WinMDI 2.9. The forward scatter (FLI-H) in the histograms (Fig 5B) demonstrated the increase in fluorescence intensity.

### Determination of pneumolysin activity by lysis of erythrocytes

*S*. *pneumoniae* D39 and corresponding *aliB*, *comDE* or double mutants were cultured in THY medium. Samples were taken at OD_600nm_ of 0.8 and 1.2, centrifuged at 3,000 g for 10 min at RT. The supernatant was collected and stored at 4°C. Fresh human erythrocytes solubilized in PBS buffer were used. The activity assay was performed as described by Benton et al., with minor modifications. A microtiter plate (Nunc) was loaded with 0.1 ml lysis buffer (10 mM DTT, 0.1% BSA in BPS buffer, pH 7.4) 0.05 ml of the corresponding culture supernatant, and 0.05 ml of the erythrocyte solution. The plate was incubated for 45 min at 37°C. As a control only buffer and erythrocyte solution was loaded. After centrifugation at 530 g at RT for 10 min the microtiter plate was analysed. Pneumolysin activity was detected by haemolysis and no erythrocyte sediment was obtained.

### Statistical analysis

All data are reported as mean ± SD unless otherwise noted. Results were statistically analysed using the unpaired two-tailed Student´s test. Kaplan-Meier survival curves were compared by the log-rank test. *P*-values for bioluminescence measurements were calculated using the unpaired, one-tailed t-test for differences between groups, while differences of one group between days were analysed by paired t-test. For animal experiments, samples were conducted under the supervision of a professional statistician during the planning period of the project. The proteomics study was considered as an explorative descriptive investigation. Based on the documented research experience in this field, a sample size of 5 was judged adequate for this approach. The virulence study was considered as a comparative experiment. Sample size calculations were done with the primary outcome parameters clinical score and the CSF leukocyte counts. The calculations were based on a multiple group comparison design using an analysis of variance (ANOVA) model, assuming 80% power, α of 0.05, and two-tailed test for statistical significance. The sample size per group needed to detect a significant difference was found to be 8 for the mutant groups and 13 for the wild type control group. Methods for statistical analysis of *in vivo* data comprised the Shapiro-Wilk normality test, the Brown–Forsythe equality of variance test, and One-Way ANOVA analysis with Tukey post-hoc test. A *P-*value <0.05 was considered to be statistically significant.

### Accession numbers

Raw data and proteomics data can be accessed via the PeptideAtlas (http://www.peptideatlas.org/).

## Supporting information

S1 Fig Generation and verification of *comDE* and *aliB* mutants in D39. (**A**) Insertion-deletion mutagenesis was carried out to inactivate *aliB* and *comDE*. *AliB* was replaced by the *cat* gene expressing chloramphenicol resistance and *comDE* was replaced by *aphA3* encoding kanamycin resistance. (**B**) Molecular analysis of *comDE*, *aliB* and *comDE/aliB* mutants by PCR. Total DNA from wild-type and corresponding mutants were used as template with primer pair aliB_1276/aliB_1277 or primer combination comDE_1224/comDE_1225 to demonstrate *aliB* (left panel) and *comDE* (right panel) inactivation. (**C**) Analysis of AliB expression in D39 wild-type and mutants. Immunoblotting was performed with polyclonal anti-AliB serum and secondary goat anti-mouse IgG conjugated with alkaline phosphatase. Detection of AliB or homologs was done with NBT/BCIP for color development. Anti-ArcB antibodies were choosen for ArcB detection as loading control. Due to high similarity of AmiA, AliA and AliB all of these proteins were detected with anti-AliB polyclonal antibodies.
(TIF)

S2 Fig Growth of pneumococcal mutants. Growth of pneumococcal wild-type and isogenic mutants in chemically-defined medium (CDM = RPMI_modi_) or in CSF from humans. Determined growth rates in CDM are: D39 (μ = 0.65 h^-1^), Δ*aliB*-mutant (μ = 0.65 h^-1^), Δ*comDE*-mutant (μ = 0.66 h^-1^), and Δ*aliB*Δ*comDE*-mutant (μ = 0.69h^-1^). In CSF the following growth rates were estimated: D39 (μ = 0.54 h^-1^), Δ*aliB*-mutant (μ = 0.49 h^-1^), Δ*comDE*-mutant (μ = 0.48 h^-1^), and Δ*aliB*Δ*comDE*-mutant (μ = 0.50 h^-1^).
(TIF)

S3 Fig Lack of ComDE and/or AliB was associated with increased motor activity and reduced weight loss in murine pneumococcal meningitis. Pneumococcal meningitis was induced by intracisternal injection of wild type *S*. *pneumoniae* D39 (n = 13) or its isogenic ComDE-deficient, AliB-deficient- or AliB-ComDE-double-deficient mutants (each mutant n = 8). Eighteen hours later, motor activity **(A)** and body weight **(B)** were determined using an open field test and a precision scale, respectively. **(A)** Mice infected with the single or double mutants showed significant increased motor activity when compared to D39 infected mice. **(B)** Mice infected with the double mutant also exhibited less pronounced weight loss than those infected with D39. In negative controls (mice injected i.c. with PBS instead of *S*. *pneumoniae*; n = 8), motor activity was 45.0 ± 9.9 fields/min, whereas weight loss was 0.3 ± 0.4%. Data are given as means ± SD. * P < 0.01, compared to mice infected with the D39 strain, using One-Way ANOVA and Tukey post-hoc test.
(TIF)

S1 Table Proteins with differential abundance between *S.p.* D39Δ*comDE* or *S.p.* D39Δ*aliB* strains and the wild-type strain. (PDF)

S2 Table Strain and plasmid list. (PDF)

S3 Table Primer list. (PDF)

S4 Table LC-MS/MS settings for analysis of samples of *in vitro* cultivated *S. pneumoniae* D39 wild-type and *S.p.* D39Δ*comDE* and *S.p.* D39Δ*aliB* mutants. (PDF)

S5 Table *In vitro* data of *S. pneumoniae* mutants from high sensitive LCMS/MS. (XLSX)

S6 Table SpectraST and in vivo data from high sensitive LCMS/MS approach. (XLSX)

## References

[1] WHO. The global burden of disease: 2004 update World Health Organization 2008.

[2] Collaborators GBDM. Global, regional, and national burden of meningitis, 1990–2016: a systematic analysis for the Global Burden of Disease Study 2016. Lancet Neurol. 2018;17(12):1061–82. 10.1016/S1474-4422(18)30387-9 30507391 PMC6234314

[3] Thigpen MC, Whitney CG, Messonnier NE, Zell ER, Lynfield R, Hadler JL, et al Bacterial meningitis in the United States, 1998–2007. N Engl J Med. 2011;364(21):2016–25. 10.1056/NEJMoa1005384 . 21612470

[4] Bijlsma MW, Brouwer MC, Kasanmoentalib ES, Kloek AT, Lucas MJ, Tanck MW, et al Community-acquired bacterial meningitis in adults in the Netherlands, 2006–14: a prospective cohort study. Lancet Infect Dis. 2016;16(3):339–47. 10.1016/S1473-3099(15)00430-2 . 26652862

[5] van de Beek D, Brouwer M, Hasbun R, Koedel U, Whitney CG, Wijdicks E. Community-acquired bacterial meningitis. Nat Rev Dis Primers. 2016;2:16074 10.1038/nrdp.2016.74 . 27808261

[6] Hupp S, Heimeroth V, Wippel C, Fortsch C, Ma J, Mitchell TJ, et al Astrocytic tissue remodeling by the meningitis neurotoxin pneumolysin facilitates pathogen tissue penetration and produces interstitial brain edema. Glia. 2012;60(1):137–46. 10.1002/glia.21256 . 21989652

[7] Wall EC, Gordon SB, Hussain S, Goonetilleke UR, Gritzfeld J, Scarborough M, et al Persistence of pneumolysin in the cerebrospinal fluid of patients with pneumococcal meningitis is associated with mortality. Clin Infect Dis. 2012;54(5):701–5. 10.1093/cid/cir926 22238165 PMC3275762

[8] Wippel C, Maurer J, Fortsch C, Hupp S, Bohl A, Ma J, et al Bacterial cytolysin during meningitis disrupts the regulation of glutamate in the brain, leading to synaptic damage. PLoS Pathog. 2013;9(6):e1003380 10.1371/journal.ppat.1003380 23785278 PMC3681734

[9] Hoegen T, Tremel N, Klein M, Angele B, Wagner H, Kirschning C, et al The NLRP3 inflammasome contributes to brain injury in pneumococcal meningitis and is activated through ATP-dependent lysosomal cathepsin B release. J Immunol. 2011;187(10):5440–51. 10.4049/jimmunol.1100790 . 22003197

[10] Ostergaard C, Brandt C, Konradsen HB, Samuelsson S. Differences in survival, brain damage, and cerebrospinal fluid cytokine kinetics due to meningitis caused by 3 different Streptococcus pneumoniae serotypes: evaluation in humans and in 2 experimental models. J Infect Dis. 2004;190(7):1212–20. Epub 2004/09/04. 10.1086/423852 . 15346330

[11] Banerjee A, Van Sorge NM, Sheen TR, Uchiyama S, Mitchell TJ, Doran KS. Activation of brain endothelium by pneumococcal neuraminidase NanA promotes bacterial internalization. Cell Microbiol. 2010;12(11):1576–88. 10.1111/j.1462-5822.2010.01490.x 20557315 PMC2943548

[12] Uchiyama S, Carlin AF, Khosravi A, Weiman S, Banerjee A, Quach D, et al The surface-anchored NanA protein promotes pneumococcal brain endothelial cell invasion. J Exp Med. 2009;206(9):1845–52. 10.1084/jem.20090386 19687228 PMC2737157

[13] Pracht D, Elm C, Gerber J, Bergmann S, Rohde M, Seiler M, et al PavA of Streptococcus pneumoniae modulates adherence, invasion, and meningeal inflammation. Infect Immun. 2005;73(5):2680–9. 10.1128/IAI.73.5.2680-2689.2005 15845469 PMC1087317

[14] Iovino F, Engelen-Lee JY, Brouwer M, van de Beek D, van der Ende A, Valls Seron M, et al pIgR and PECAM-1 bind to pneumococcal adhesins RrgA and PspC mediating bacterial brain invasion. J Exp Med. 2017;214(6):1619–30. 10.1084/jem.20161668 28515075 PMC5461002

[15] Kadioglu A, Weiser JN, Paton JC, Andrew PW. The role of Streptococcus pneumoniae virulence factors in host respiratory colonization and disease. Nat Rev Microbiol. 2008;6(4):288–301. Epub 2008/03/15. 10.1038/nrmicro1871 . 18340341

[16] Bergmann S, Hammerschmidt S. Versatility of pneumococcal surface proteins. Microbiology. 2006;152(Pt 2):295–303. Epub 2006/01/27. 10.1099/mic.0.28610-0 . 16436417

[17] Krone CL, van de Groep K, Trzcinski K, Sanders EA, Bogaert D. Immunosenescence and pneumococcal disease: an imbalance in host-pathogen interactions. Lancet Respir Med. 2014;2(2):141–53. 10.1016/S2213-2600(13)70165-6 . 24503269

[18] Hava DL, Camilli A. Large-scale identification of serotype 4 Streptococcus pneumoniae virulence factors. Mol Microbiol. 2002;45(5):1389–406. 12207705 PMC2788772

[19] Orihuela CJ, Radin JN, Sublett JE, Gao G, Kaushal D, Tuomanen EI. Microarray analysis of pneumococcal gene expression during invasive disease. Infect Immun. 2004;72(10):5582–96. 10.1128/IAI.72.10.5582-5596.2004 15385455 PMC517545

[20] Kulohoma BW, Cornick JE, Chaguza C, Yalcin F, Harris SR, Gray KJ, et al Comparative Genomic Analysis of Meningitis- and Bacteremia-Causing Pneumococci Identifies a Common Core Genome. Infect Immun. 2015;83(10):4165–73. 10.1128/IAI.00814-15 26259813 PMC4567637

[21] Lees JA, Kremer PH, Manso AS, Croucher NJ, Ferwerda B, Seron MV, et al Large scale genomic analysis shows no evidence for pathogen adaptation between the blood and cerebrospinal fluid niches during bacterial meningitis. Microb Genom. 2017;3(1):e000103 10.1099/mgen.0.000103 28348877 PMC5361624

[22] Mahdi LK, Van der Hoek MB, Ebrahimie E, Paton JC, Ogunniyi AD. Characterization of Pneumococcal Genes Involved in Bloodstream Invasion in a Mouse Model. PLoS One. 2015;10(11):e0141816 10.1371/journal.pone.0141816 26539717 PMC4634996

[23] Molzen TE, Burghout P, Bootsma HJ, Brandt CT, van der Gaast-de Jongh CE, Eleveld MJ, et al Genome-wide identification of Streptococcus pneumoniae genes essential for bacterial replication during experimental meningitis. Infect Immun. 2011;79(1):288–97. 10.1128/IAI.00631-10 21041497 PMC3019918

[24] Ogunniyi AD, Mahdi LK, Trappetti C, Verhoeven N, Mermans D, Van der Hoek MB, et al Identification of genes that contribute to the pathogenesis of invasive pneumococcal disease by in vivo transcriptomic analysis. Infect Immun. 2012;80(9):3268–78. 10.1128/IAI.00295-12 22778095 PMC3418729

[25] Michalik S, Depke M, Murr A, Gesell Salazar M, Kusebauch U, Sun Z, et al A global Staphylococcus aureus proteome resource applied to the in vivo characterization of host-pathogen interactions. Sci Rep. 2017;7(1):9718 10.1038/s41598-017-10059-w 28887440 PMC5591248

[26] Schmidt F, Lueking A, Nordhoff E, Gobom J, Klose J, Seitz H, et al Generation of minimal protein identifiers of proteins from two-dimensional gels and recombinant proteins. Electrophoresis. 2002;23(4):621–5. 10.1002/1522-2683(200202)23:4<621::AID-ELPS621>3.0.CO;2-J . 11870774

[27] Vizcaino JA, Cote RG, Csordas A, Dianes JA, Fabregat A, Foster JM, et al The PRoteomics IDEntifications (PRIDE) database and associated tools: status in 2013. Nucleic Acids Res. 2013;41(Database issue):D1063–9. 10.1093/nar/gks1262 23203882 PMC3531176

[28] Desiere F, Deutsch EW, King NL, Nesvizhskii AI, Mallick P, Eng J, et al The PeptideAtlas project. Nucleic Acids Res. 2006;34(Database issue):D655–8. 10.1093/nar/gkj040 16381952 PMC1347403

[29] Lam H, Deutsch EW, Eddes JS, Eng JK, Stein SE, Aebersold R. Building consensus spectral libraries for peptide identification in proteomics. Nat Methods. 2008;5(10):873–5. 10.1038/nmeth.1254 18806791 PMC2637392

[30] Alloing G, de Philip P, Claverys JP. Three highly homologous membrane-bound lipoproteins participate in oligopeptide transport by the Ami system of the gram-positive Streptococcus pneumoniae. J Mol Biol. 1994;241(1):44–58. 10.1006/jmbi.1994.1472 . 8051706

[31] Woehrl B, Brouwer MC, Murr C, Heckenberg SG, Baas F, Pfister HW, et al Complement component 5 contributes to poor disease outcome in humans and mice with pneumococcal meningitis. J Clin Invest. 2011;121(10):3943–53. 10.1172/JCI57522 21926466 PMC3195471

[32] Brouwer MC, Meijers JC, Baas F, van der Ende A, Pfister HW, Giese A, et al Plasminogen activator inhibitor-1 influences cerebrovascular complications and death in pneumococcal meningitis. Acta Neuropathol. 2014;127(4):553–64. 10.1007/s00401-013-1216-4 . 24248324

[33] Schulz C, Gierok P, Petruschka L, Lalk M, Mader U, Hammerschmidt S. Regulation of the arginine deiminase system by ArgR2 interferes with arginine metabolism and fitness of Streptococcus pneumoniae. MBio. 2014;5(6). Epub 2014/12/30. 10.1128/mBio.01858-14 25538192 PMC4278536

[34] Alloing G, Granadel C, Morrison DA, Claverys JP. Competence pheromone, oligopeptide permease, and induction of competence in Streptococcus pneumoniae. Mol Microbiol. 1996;21(3):471–8. 10.1111/j.1365-2958.1996.tb02556.x 8866471

[35] Yang XY, He K, Du G, Wu X, Yu G, Pan Y, et al Integrated Translatomics with Proteomics to Identify Novel Iron-Transporting Proteins in Streptococcus pneumoniae. Front Microbiol. 2016;7:78 10.3389/fmicb.2016.00078 26870030 PMC4738293

[36] Doran KS, Fulde M, Gratz N, Kim BJ, Nau R, Prasadarao N, et al Host-pathogen interactions in bacterial meningitis. Acta Neuropathol. 2016;131(2):185–209. 10.1007/s00401-015-1531-z 26744349 PMC4713723

[37] Huhmer AF, Biringer RG, Amato H, Fonteh AN, Harrington MG. Protein analysis in human cerebrospinal fluid: Physiological aspects, current progress and future challenges. Dis Markers. 2006;22(1–2):3–26. 10.1155/2006/158797 16410649 PMC3850820

[38] Spector R, Robert Snodgrass S, Johanson CE. A balanced view of the cerebrospinal fluid composition and functions: Focus on adult humans. Exp Neurol. 2015;273:57–68. 10.1016/j.expneurol.2015.07.027 . 26247808

[39] Wright BL, Lai JT, Sinclair AJ. Cerebrospinal fluid and lumbar puncture: a practical review. J Neurol. 2012;259(8):1530–45. 10.1007/s00415-012-6413-x . 22278331

[40] Arevalo CE, Barnes PF, Duda M, Leedom JM. Cerebrospinal fluid cell counts and chemistries in bacterial meningitis. South Med J. 1989;82(9):1122–7. 10.1097/00007611-198909000-00016 . 2772683

[41] Lindquist L, Linne T, Hansson LO, Kalin M, Axelsson G. Value of cerebrospinal fluid analysis in the differential diagnosis of meningitis: a study in 710 patients with suspected central nervous system infection. Eur J Clin Microbiol Infect Dis. 1988;7(3):374–80. . 3137038 10.1007/BF01962340

[42] Kerr AR, Adrian PV, Estevao S, de Groot R, Alloing G, Claverys JP, et al The Ami-AliA/AliB permease of Streptococcus pneumoniae is involved in nasopharyngeal colonization but not in invasive disease. Infect Immun. 2004;72(7):3902–6. 10.1128/IAI.72.7.3902-3906.2004 15213133 PMC427416

[43] Kowalko JE, Sebert ME. The Streptococcus pneumoniae competence regulatory system influences respiratory tract colonization. Infect Immun. 2008;76(7):3131–40. 10.1128/IAI.01696-07 18443092 PMC2446691

[44] Hirst RA, Gosai B, Rutman A, Guerin CJ, Nicotera P, Andrew PW, et al Streptococcus pneumoniae deficient in pneumolysin or autolysin has reduced virulence in meningitis. J Infect Dis. 2008;197(5):744–51. 10.1086/527322 . 18260758

[45] Bradshaw JL, Pipkins HR, Keller LE, Pendarvis JK, McDaniel LS. Mucosal Infections and Invasive Potential of Nonencapsulated Streptococcus pneumoniae Are Enhanced by Oligopeptide Binding Proteins AliC and AliD. MBio. 2018;9(1). 10.1128/mBio.02097-17 29339428 PMC5770551

[46] Hilty M, Wuthrich D, Salter SJ, Engel H, Campbell S, Sa-Leao R, et al Global phylogenomic analysis of nonencapsulated Streptococcus pneumoniae reveals a deep-branching classic lineage that is distinct from multiple sporadic lineages. Genome Biol Evol. 2014;6(12):3281–94. 10.1093/gbe/evu263 25480686 PMC4986459

[47] Nasher F, Forster S, Yildirim EC, Grandgirard D, Leib SL, Heller M, et al Foreign peptide triggers boost in pneumococcal metabolism and growth. BMC Microbiol. 2018;18(1):23 10.1186/s12866-018-1167-y 29580217 PMC5870813

[48] Gomez-Baena G, Bennett RJ, Martinez-Rodriguez C, Wnek M, Laing G, Hickey G, et al Quantitative Proteomics of Cerebrospinal Fluid in Paediatric Pneumococcal Meningitis. Sci Rep. 2017;7(1):7042 10.1038/s41598-017-07127-6 28765563 PMC5539295

[49] Depke M, Michalik S, Rabe A, Surmann K, Brinkmann L, Jehmlich N, et al A peptide resource for the analysis of Staphylococcus aureus in host-pathogen interaction studies. Proteomics. 2015;15(21):3648–61. 10.1002/pmic.201500091 26224020 PMC4886865

[50] Aprianto R, Slager J, Holsappel S, Veening JW. High-resolution analysis of the pneumococcal transcriptome under a wide range of infection-relevant conditions. Nucleic Acids Res. 2018;46(19):9990–10006. 10.1093/nar/gky750 30165663 PMC6212715

[51] Ibrahim YM, Kerr AR, McCluskey J, Mitchell TJ. Role of HtrA in the virulence and competence of Streptococcus pneumoniae. Infect Immun. 2004;72(6):3584–91. 10.1128/IAI.72.6.3584-3591.2004 15155668 PMC415679

[52] Pestova EV, Havarstein LS, Morrison DA. Regulation of competence for genetic transformation in Streptococcus pneumoniae by an auto-induced peptide pheromone and a two-component regulatory system. Mol Microbiol. 1996;21(4):853–62. 10.1046/j.1365-2958.1996.501417.x . 8878046

[53] Sebert ME, Patel KP, Plotnick M, Weiser JN. Pneumococcal HtrA protease mediates inhibition of competence by the CiaRH two-component signaling system. J Bacteriol. 2005;187(12):3969–79. 10.1128/JB.187.12.3969-3979.2005 15937159 PMC1151733

[54] Zhu L, Lin J, Kuang Z, Vidal JE, Lau GW. Deletion analysis of Streptococcus pneumoniae late competence genes distinguishes virulence determinants that are dependent or independent of competence induction. Mol Microbiol. 2015;97(1):151–65. 10.1111/mmi.13016 25846124 PMC4536566

[55] Havarstein LS. Identification of a competence regulon in Streptococcus pneumoniae by genomic analysis. Trends Microbiol. 1998;6(8):297–9; discussion 9–300. . 9746937 10.1016/s0966-842x(98)01328-6

[56] Gomez-Mejia A, Gamez G, Hammerschmidt S. Streptococcus pneumoniae two-component regulatory systems: The interplay of the pneumococcus with its environment. Int J Med Microbiol. 2018;308(6):722–37. 10.1016/j.ijmm.2017.11.012 . 29221986

[57] Lau GW, Haataja S, Lonetto M, Kensit SE, Marra A, Bryant AP, et al A functional genomic analysis of type 3 Streptococcus pneumoniae virulence. Mol Microbiol. 2001;40(3):555–71. . 11359563 10.1046/j.1365-2958.2001.02335.x

[58] Johnsborg O, Havarstein LS. Regulation of natural genetic transformation and acquisition of transforming DNA in Streptococcus pneumoniae. FEMS Microbiol Rev. 2009;33(3):627–42. 10.1111/j.1574-6976.2009.00167.x . 19396959

[59] Dagkessamanskaia A, Moscoso M, Henard V, Guiral S, Overweg K, Reuter M, et al Interconnection of competence, stress and CiaR regulons in Streptococcus pneumoniae: competence triggers stationary phase autolysis of ciaR mutant cells. Mol Microbiol. 2004;51(4):1071–86. . 14763981 10.1111/j.1365-2958.2003.03892.x

[60] Slager J, Aprianto R, Veening JW. Deep genome annotation of the opportunistic human pathogen Streptococcus pneumoniae D39. Nucleic Acids Res. 2018;46(19):9971–89. 10.1093/nar/gky725 30107613 PMC6212727

[61] Domenech A, Slager J, Veening JW. Antibiotic-Induced Cell Chaining Triggers Pneumococcal Competence by Reshaping Quorum Sensing to Autocrine-Like Signaling. Cell Rep. 2018;25(9):2390–400 e3. 10.1016/j.celrep.2018.11.007 30485808 PMC6289044

[62] Prudhomme M, Attaiech L, Sanchez G, Martin B, Claverys JP. Antibiotic stress induces genetic transformability in the human pathogen Streptococcus pneumoniae. Science. 2006;313(5783):89–92. 10.1126/science.1127912 . 16825569

[63] Slager J, Kjos M, Attaiech L, Veening JW. Antibiotic-induced replication stress triggers bacterial competence by increasing gene dosage near the origin. Cell. 2014;157(2):395–406. 10.1016/j.cell.2014.01.068 . 24725406

[64] Stevens KE, Chang D, Zwack EE, Sebert ME. Competence in Streptococcus pneumoniae is regulated by the rate of ribosomal decoding errors. MBio. 2011;2(5). 10.1128/mBio.00071-11 21933920 PMC3175624

[65] Solomon JM, Magnuson R, Srivastava A, Grossman AD. Convergent sensing pathways mediate response to two extracellular competence factors in Bacillus subtilis. Genes Dev. 1995;9(5):547–58. 10.1101/gad.9.5.547 . 7698645

[66] Hathaway LJ, Stutzmann Meier P, Battig P, Aebi S, Muhlemann K. A homologue of aliB is found in the capsule region of nonencapsulated Streptococcus pneumoniae. J Bacteriol. 2004;186(12):3721–9. 10.1128/JB.186.12.3721-3729.2004 15175285 PMC419944

[67] Hartel T, Eylert E, Schulz C, Petruschka L, Gierok P, Grubmuller S, et al Characterization of central carbon metabolism of Streptococcus pneumoniae by isotopologue profiling. J Biol Chem. 2012;287(6):4260–74. 10.1074/jbc.M111.304311 22167202 PMC3281726

[68] Shepherd J, Ibba M. Relaxed substrate specificity leads to extensive tRNA mischarging by Streptococcus pneumoniae class I and class II aminoacyl-tRNA synthetases. MBio. 2014;5(5):e01656–14. 10.1128/mBio.01656-14 25205097 PMC4173786

[69] Chiavolini D, Pozzi G, Ricci S. Animal models of Streptococcus pneumoniae disease. Clin Microbiol Rev. 2008;21(4):666–85. 10.1128/CMR.00012-08 18854486 PMC2570153

[70] Saleh M, Bartual SG, Abdullah MR, Jensch I, Asmat TM, Petruschka L, et al Molecular architecture of Streptococcus pneumoniae surface thioredoxin-fold lipoproteins crucial for extracellular oxidative stress resistance and maintenance of virulence. EMBO Mol Med. 2013;5(12):1852–70. Epub 2013/10/19. 10.1002/emmm.201202435 24136784 PMC3914529

[71] Hohne C, Wenzel M, Angele B, Hammerschmidt S, Hacker H, Klein M, et al High mobility group box 1 prolongs inflammation and worsens disease in pneumococcal meningitis. Brain. 2013;136(Pt 6):1746–59. 10.1093/brain/awt064 . 23518713

[72] Keller A, Eng J, Zhang N, Li XJ, Aebersold R. A uniform proteomics MS/MS analysis platform utilizing open XML file formats. Mol Syst Biol. 2005;1:2005 0017. 10.1038/msb4100024 16729052 PMC1681455

[73] Cox J, Mann M. MaxQuant enables high peptide identification rates, individualized p.p.b.-range mass accuracies and proteome-wide protein quantification. Nat Biotechnol. 2008;26(12):1367–72. 10.1038/nbt.1511 . 19029910

[74] Overbeek R, Olson R, Pusch GD, Olsen GJ, Davis JJ, Disz T, et al The SEED and the Rapid Annotation of microbial genomes using Subsystems Technology (RAST). Nucleic Acids Res. 2014;42(Database issue):D206–14. 10.1093/nar/gkt1226 24293654 PMC3965101

[75] Bernhardt J, Funke S, Hecker M, Sieburg J. Visualizing gene expression data via Voronoi Treemaps. *Sixth International Symposium on Voronoi Diagrams*; 20092009. p. 233–44.

[76] Halfmann A, Hakenbeck R, Bruckner R. A new integrative reporter plasmid for Streptococcus pneumoniae. FEMS Microbiol Lett. 2007;268(2):217–24. 10.1111/j.1574-6968.2006.00584.x . 17328748

[77] Abdullah MR, Gutierrez-Fernandez J, Pribyl T, Gisch N, Saleh M, Rohde M, et al Structure of the pneumococcal l,d-carboxypeptidase DacB and pathophysiological effects of disabled cell wall hydrolases DacA and DacB. Mol Microbiol. 2014;93(6):1183–206. Epub 2014/07/26. 10.1111/mmi.12729 . 25060741

