# Supplementary figures and images for: In vivo proteomics identifies the competence regulon and AliB oligopeptide transporter as pathogenic factors in pneumococcal meningitis
Source: PLoS Pathog. 2019 Jul 29;15(7):e1007987. doi: 10.1371/journal.ppat.1007987 (PMC6687184; doi:10.1371/journal.ppat.1007987)

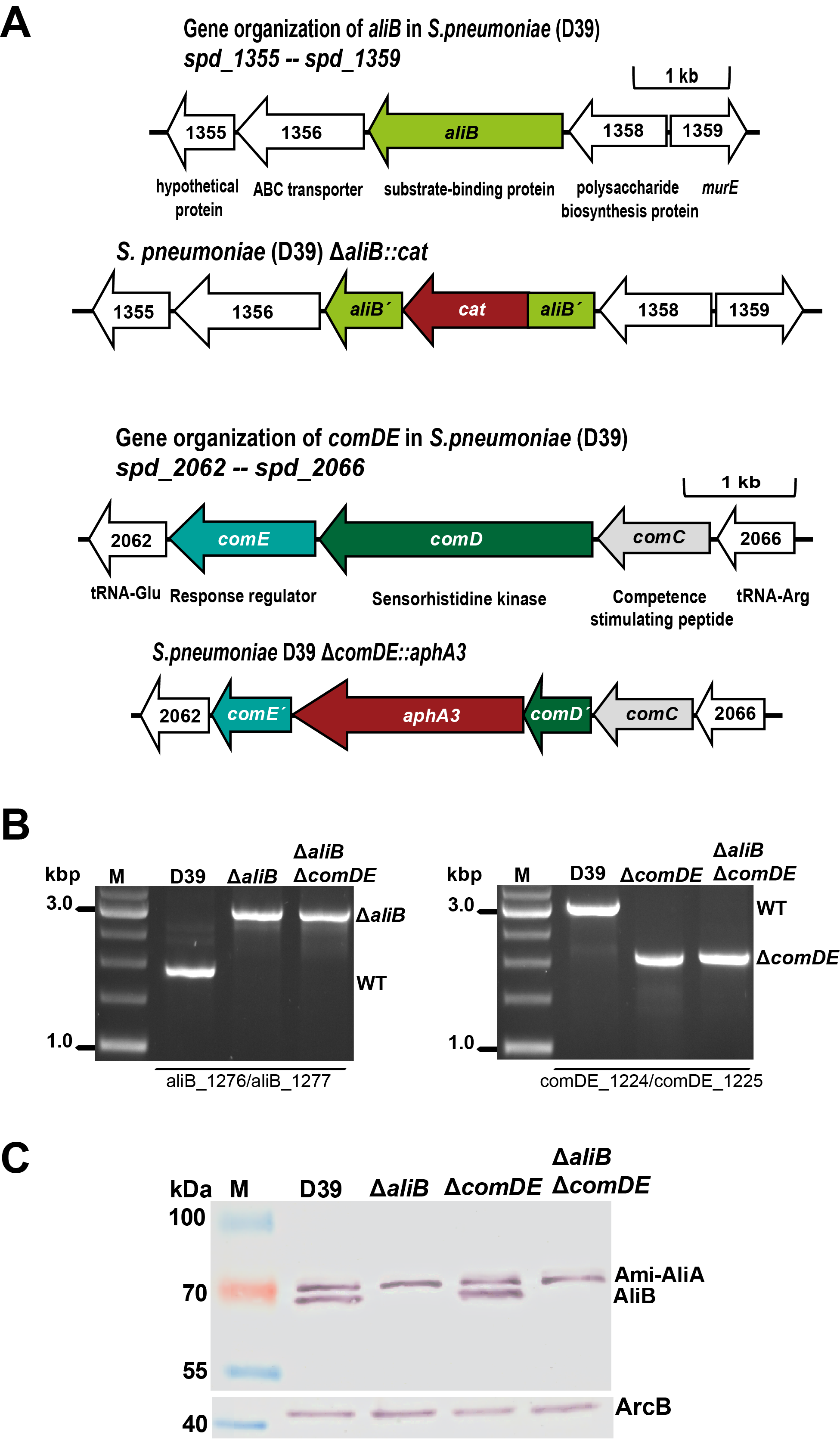

Supplement: S1 Fig — (A) Insertion-deletion mutagenesis was carried out to inactivate aliB and comDE. AliB was replaced by the cat gene expressing chloramphenicol resistance and comDE was replaced by aphA3 encoding kanamycin resistance. (B) Molecular analysis of comDE, aliB and comDE/aliB mutants by PCR. Total DNA from wild-type and corresponding mutants were used as template with primer pair aliB_1276/aliB_1277 or primer combination comDE_1224/comDE_1225 to demonstrate aliB (left panel) and comDE (right panel) inactivation. (C) Analysis of AliB expression in D39 wild-type and mutants. Immunoblotting was performed with polyclonal anti-AliB serum and secondary goat anti-mouse IgG conjugated with alkaline phosphatase. Detection of AliB or homologs was done with NBT/BCIP for color development. Anti-ArcB antibodies were choosen for ArcB detection as loading control. Due to high similarity of AmiA, AliA and AliB all of these proteins were detected with anti-AliB polyclonal antibodies. (TIF) [file ppat.1007987.s001.tif]

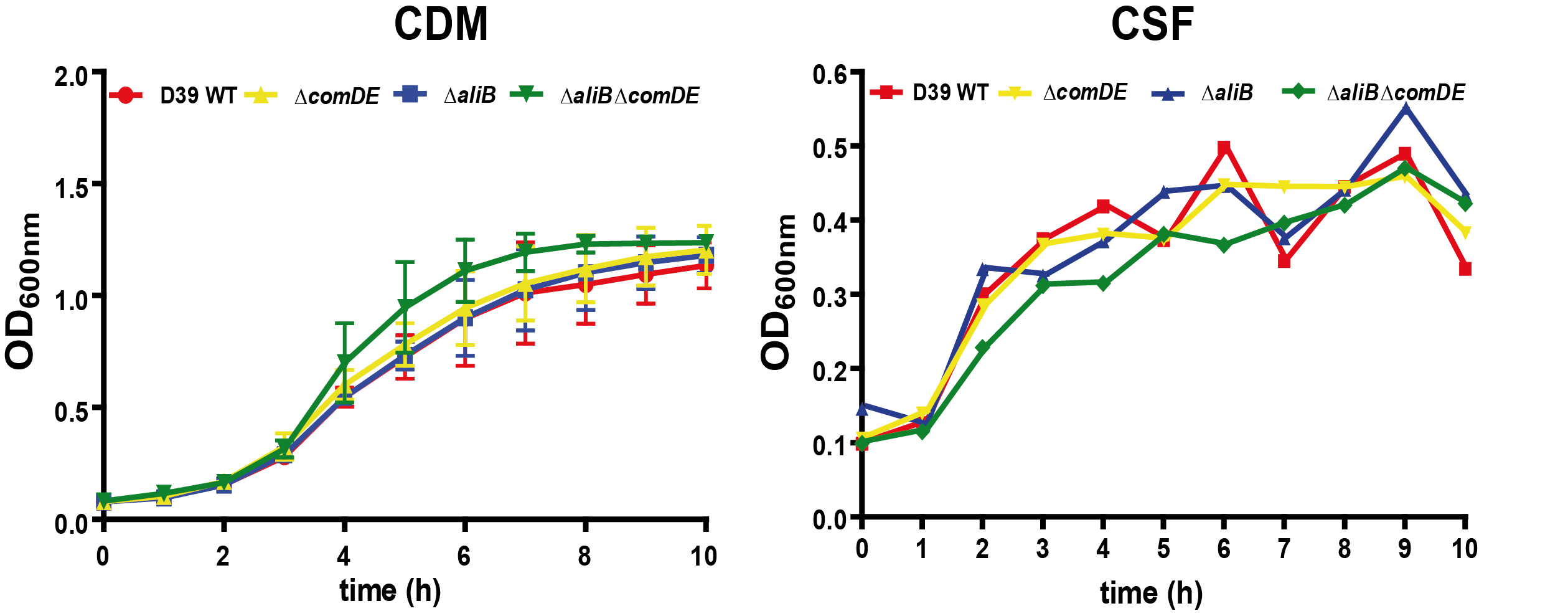

Supplement: S2 Fig — Growth of pneumococcal wild-type and isogenic mutants in chemically-defined medium (CDM = RPMImodi) or in CSF from humans. Determined growth rates in CDM are: D39 (μ = 0.65 h-1), ΔaliB-mutant (μ = 0.65 h-1), ΔcomDE-mutant (μ = 0.66 h-1), and ΔaliBΔcomDE-mutant (μ = 0.69h-1). In CSF the following growth rates were estimated: D39 (μ = 0.54 h-1), ΔaliB-mutant (μ = 0.49 h-1), ΔcomDE-mutant (μ = 0.48 h-1), and ΔaliBΔcomDE-mutant (μ = 0.50 h-1). (TIF) [file ppat.1007987.s002.tif]

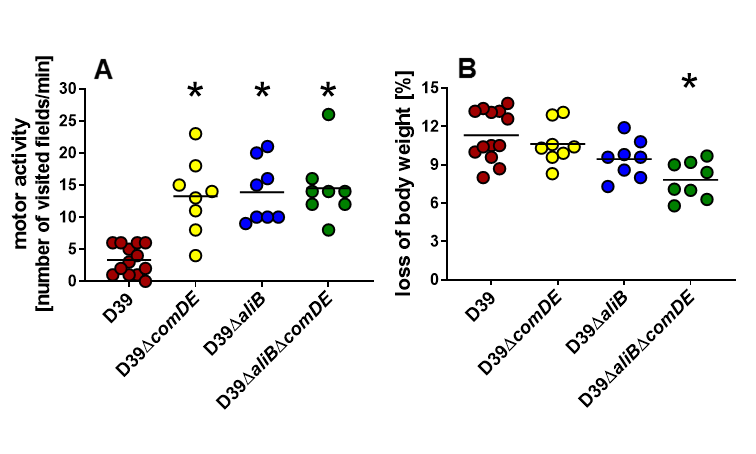

Supplement: S3 Fig — Pneumococcal meningitis was induced by intracisternal injection of wild type S. pneumoniae D39 (n = 13) or its isogenic ComDE-deficient, AliB-deficient- or AliB-ComDE-double-deficient mutants (each mutant n = 8). Eighteen hours later, motor activity (A) and body weight (B) were determined using an open field test and a precision scale, respectively. (A) Mice infected with the single or double mutants showed significant increased motor activity when compared to D39 infected mice. (B) Mice infected with the double mutant also exhibited less pronounced weight loss than those infected with D39. In negative controls (mice injected i.c. with PBS instead of S. pneumoniae; n = 8), motor activity was 45.0 ± 9.9 fields/min, whereas weight loss was 0.3 ± 0.4%. Data are given as means ± SD. * P < 0.01, compared to mice infected with the D39 strain, using One-Way ANOVA and Tukey post-hoc test. (TIF) [file ppat.1007987.s003.tif]
