# Supplementary material for: In vivo proteomics identifies the competence regulon and AliB oligopeptide transporter as pathogenic factors in pneumococcal meningitis
Source: PLoS Pathog. 2019 Jul 29;15(7):e1007987. doi: 10.1371/journal.ppat.1007987 (PMC6687184; doi:10.1371/journal.ppat.1007987)
Supplement: S1 Table — (PDF) [file ppat.1007987.s004.pdf]

Table S1: Proteins with differential abundance between *S.p.* D39Δ*comDE* or *S.p.* D39Δ*aliB* strains and the wild-type strain. Proteins with a p-value of 0.05 or less and an absolute fold change of at least 1.5 were regarded as significantly regulated. Furthermore, proteins, which were quantified in all replicates of one condition and not detected in all replicates of the comparison condition (mutant or wt, early or mid-exponential phase), were classified as regulated with an on-off effect. In addition, less stringent criteria, which allowed the quantification of a protein in one of four replicates in one condition while requiring quantification in all replicates of the other condition and an absolute fold change of at least 2, were applied for further defining on-off regulated proteins.

a. Regulated proteins (n=24) in the comparison of *S.p.* D39Δ*comDE* strain and *S.p.* D39 wild-type strain in early exponential growth phase.

			**Δ*comDE* *vs.* wt**				**Δ*aliB* *vs.* wt**			
			**early exp. growth phase**		**mid-exp. growth phase**		**early exp. growth phase**		**mid-exp. growth phase**	
**identifier**	**gene**	**description**	**fold change**	**sign.[a]**	**fold change**	**sign.[a]**	**fold change**	**sign.[a]**	**fold change**	**sign.[a]**
spd_0102 | spd_rs00535	*sdhA*	L-serine dehydratase, iron-sulfur-dependent subunit alpha (EC:4.3.1.17)	**1.56**	**s.**	1.13	n.s.	1.13	n.s.	1.41	N/A
spd_0114 | spd_rs00600	namex 120	hypothetical protein	**-4.09**	**off**	1.17	n.s.	N/A	off	-3.30	N/A
spd_0115 | spd_rs00605	namex 121	hypothetical protein	**-2.70**	**off**	1.01	n.s.	N/A	off	N/A	off
spd_0203 | spd_rs01095	*rplN*	5S ribosomal protein L14	**1.55**	**s.**	1.30	s.	1.14	n.s.	1.15	n.s.
spd_0308 | spd_rs01645	*clpL*	ATP-dependent Clp protease, ATP-binding subunit	**1.69**	**s.**	1.54	s.	1.76	s.	2.72	s.
spd_0489 | spd_rs02610	namex 520	hypothetical protein	**1.50**	**s.**	1.20	N/A	1.19	n.s.	-1.03	n.s.
spd_0608 | spd_rs03250	*pyrF*	orotidine 5-phosphate decarboxylase (EC:4.1.1.23)	**2.78**	**s.**	1.28	N/A	2.08	n.s.	1.23	n.s.
spd_0609 | spd_rs03255	*pyrE*	orotate phosphoribosyl-transferase (EC:2.4.2.1)	**1.99**	**s.**	-1.15	n.s.	1.36	n.s.	1.01	n.s.
spd_0696 | spd_rs03690	namex 735	MutT/nudix family protein	**N/A**	**on**	1.09	N/A	N/A	on	1.23	N/A
spd_0852 | spd_rs04545	*pyrDb*	dihydroorotate dehydrogenase 1B (EC:1.3.98.1)	**2.65**	**s.**	-1.01	n.s.	2.03	n.s.	1.02	n.s.
spd_1131 | spd_rs05980	*carB*	carbamoyl phosphate synthase large subunit (EC:6.3.5.5)	**2.35**	**s.**	1.09	n.s.	1.71	n.s.	1.16	n.s.
spd_1133 | spd_rs05990	*pyrB*	aspartate carbamoyl-transferase catalytic subunit (EC:2.1.3.2)	**1.99**	**s.**	1.18	n.s.	1.59	n.s.	1.17	s.
spd_1134 | spd_rs05995	*pyrR*	bifunctional pyrimidine regulatory protein PyrR/uracil phospho-ribosyltransferase (EC:2.4.2.9)	**1.87**	**s.**	1.29	n.s.	1.64	s.	1.21	n.s.
spd_1334 | spd_rs07025	*atpC*	FF1 ATP synthase subunit epsilon (EC:3.6.3.14)	**1.55**	**s.**	1.13	n.s.	1.29	s.	-1.12	n.s.
spd_1485 | spd_rs07835	*recR*	recombination protein RecR	**N/A**	**on**	1.46	N/A	N/A	N/A	1.25	N/A
spd_1667 | spd_rs08820	*amiF*	oligopeptide ABC transporter ATP-binding protein AmiF	**-1.53**	**s.**	-1.50	s.	-1.59	s.	-1.27	s.
spd_1668 | spd_rs08825	*amiE*	oligopeptide ABC transporter ATP-binding protein AmiE	**-1.82**	**s.**	-1.95	s.	-1.92	s.	-1.74	s.
spd_1669 | spd_rs08830	*amiD*	oligopeptide ABC transporter permease AmiD	**-2.92**	**s.**	-2.93	s.	-2.04	s.	-1.97	s.

spd_1670 | spd_rs08835	*amiC*	oligopeptide ABC transporter permease AmiC	**-2.83**	**s.**	-3.16	s.	-2.42	s.	-2.43	s.
spd_1793 | spd_rs09475	namex 1888	universal stress protein family protein	**1.76**	**s.**	1.30	n.s.	-1.19	n.s.	1.28	n.s.
spd_1848 | spd_rs09755	namex 1944	hypothetical protein	**1.52**	**s.**	1.13	n.s.	1.16	n.s.	1.19	N/A
spd_1908 | spd_rs10050	*rr4*	response regulator	**1.67**	**s.**	1.35	n.s.	1.41	n.s.	1.30	n.s.
spd_2000 | spd_rs10505	*adcR*	adc operon repressor AdcR	**N/A**	**on**	1.30	n.s.	N/A	N/A	1.41	n.s.
spd_2063 | spd_rs10830	*comE*	response regulator ComE	**N/A**	**off**	N/A	off	-1.02	0.83	17.05	n.s.

[a] significance level or on-off effect (s. – significant with p ≤ 0.05; n.s. – not significant with p > 0.05; N/A – p-value not available; on – on-off effect: quantified only in mutant; off – on-off effect: not quantified in mutant)
Because of missing intensity values in the proteomics data set, not all fold change values could be calculated. Those are indicated by „N/A“.

b. Regulated proteins (n=11) in the comparison of *S.p.* D39Δ*comDE* strain and *S.p.* D39 wild-type strain in mid-exponential growth phase.

			**Δ*comDE* *vs.* wt**				**Δ*aliB* *vs.* wt**			
			**early exp. growth phase**		**mid-exp. growth phase**		**early exp. growth phase**		**mid-exp. growth phase**	
**identifier**	**gene**	**description**	**fold change**	**sign.[a]**	**fold change**	**sign.[a]**	**fold change**	**sign.[a]**	**fold change**	**sign.[a]**
spd_0308 | spd_rs01645	*clpL*	ATP-dependent Clp protease, ATP-binding subunit	1.69	s.	**1.54**	**s.**	1.76	s.	2.72	s.
spd_0697 | spd_rs03695	namex 736	GNAT family acetyltransferase	N/A	N/A	**N/A**	**on**	N/A	N/A	N/A	on
spd_0989 | spd_rs05280	*rplU*	5S ribosomal protein L21	1.40	s.	**1.53**	**s.**	1.13	n.s.	1.24	n.s.
spd_1621 | spd_rs08570	namex 1707	ABC transporter ATP-binding protein/ permease	-1.17	s.	**-1.66**	**s.**	1.04	n.s.	-1.10	n.s.
spd_1622 | spd_rs08575	namex 1708	ABC transporter ATP-binding protein/ permease	-1.07	n.s.	**-1.51**	**s.**	1.09	n.s.	-1.13	n.s.
spd_1668 | spd_rs08825	*amiE*	oligopeptide ABC transporter ATP-binding protein AmiE	-1.82	s.	**-1.95**	**s.**	-1.92	s.	-1.74	s.
spd_1669 | spd_rs08830	*amiD*	oligopeptide ABC transporter permease AmiD	-2.92	s.	**-2.93**	**s.**	-2.04	s.	-1.97	s.
spd_1670 | spd_rs08835	*amiC*	oligopeptide ABC transporter permease AmiC	-2.83	s.	**-3.16**	**s.**	-2.42	s.	-2.43	s.
spd_1671 | spd_rs08840	*amiA*	oligopeptide ABC transporter oligopeptide-binding protein AmiA	-959.64	off	**N/A**	**off**	-2036.0	off	N/A	off
spd_1900 | spd_rs10010	namex 1995	ABC transporter ATP-binding protein/ permease	-1.03	n.s.	**-1.52**	**s.**	1.05	n.s.	-1.20	n.s.
spd_2063 | spd_rs10830	*comE*	response regulator ComE	N/A	off	**N/A**	**off**	-1.02	n.s.	17.05	n.s.

[a] significance level or on-off effect (s. – significant with p ≤ 0.05; n.s. – not significant with p > 0.05; N/A – p-value not available; on – on-off effect: quantified only in mutant; off – on-off effect: not quantified in mutant)
Because of missing intensity values in the proteomics data set, not all fold change values could be calculated. Those are indicated by „N/A“.

c. Regulated proteins (n=33) in the comparison of *S.p.* D39Δ*aliB* strain and *S.p.* D39 wild-type strain in early exponential growth phase.

			Δ*comDE vs.* wt				Δ*aliB vs.* wt			
			early exp. growth phase		mid-exp. growth phase		early exp. growth phase		mid-exp. growth phase	
identifier	gene	description	fold change	sign.[a]	fold change	sign.[a]	fold change	sign.[a]	fold change	sign.[a]
spd_0052 | spd_rs00280	namex 56	phosphoribosylformyl-glycinamidine synthase (EC:6.3.5.3)	-2.43	n.s.	-1.14	n.s.	**-3.83**	**s.**	-1.75	n.s.
spd_0059 | spd_rs00315	*purE*	phosphoribosylamino-imidazole carboxylase catalytic subunit (EC:4.1.1.21)	-1.35	n.s.	1.28	n.s.	**-2.35**	**s.**	-1.23	n.s.
spd_0109 | spd_rs00570	namex 114	amino acid ABC transporter periplasmic amino acid-binding protein	N/A	N/A	N/A	N/A	**363.52**	**on**	N/A	on
spd_0110 | spd_rs00575	*argG*	argininosuccinate synthase (EC:6.3.4.5)	N/A	N/A	N/A	N/A	**N/A**	**on**	N/A	on
spd_0111 | spd_rs00580	*argH*	argininosuccinate lyase (EC:4.3.2.1)	N/A	N/A	N/A	N/A	**N/A**	**on**	N/A	on
spd_0114 | spd_rs00600	namex 120	hypothetical protein	-4.09	off	1.17	n.s.	**N/A**	**off**	-3.30	#NV
spd_0115 | spd_rs00605	namex 121	hypothetical protein	-2.70	off	1.01	n.s.	**N/A**	**off**	N/A	off
spd_0260 | spd_rs01390	*rsuA-1*	ribosomal small subunit pseudouridine synthase A	1.15	n.s.	1.02	N/A	**N/A**	**off**	-1.06	N/A
spd_0308 | spd_rs01645	*clpL*	ATP-dependent Clp protease, ATP-binding subunit	1.69	s.	1.54	s.	**1.76**	**s.**	2.72	s.
spd_0661 | spd_rs03520	*exp5*	PTS system transporter subunit IIABC (EC:2.7.1.69)	1.02	n.s.	-1.72	n.s.	**2.14**	**s.**	2.39	s.
spd_0696 | spd_rs03690	namex 735	MutT/nudix family protein	N/A	on	1.09	N/A	**N/A**	**on**	1.23	N/A
spd_0719 | spd_rs03805	namex 758	amino acid ABC transporter permease	1.04	n.s.	-1.29	n.s.	**1.91**	**s.**	1.76	s.
spd_0721 | spd_rs03815	*folD*	bifunctional 5,1-methylene-tetrahydro-folate dehydrogenase/ 5,1-methylene-tetra-hydrofolate cyclo-hydrolase	-1.08	n.s.	-1.13	n.s.	**1.76**	**s.**	1.82	s.
spd_0722 | spd_rs03820	namex 761	YjeF-like protein	N/A	N/A	-1.24	N/A	**N/A**	**on**	1.22	N/A
spd_0981 | spd_rs05230	namex 1042	hypothetical protein	N/A	N/A	N/A	N/A	**N/A**	**on**	N/A	N/A
spd_1037 | spd_rs05515	namex 1099	histidine triad protein	-1.48	n.s.	-1.16	n.s.	**1.61**	**s.**	1.03	n.s.
spd_1134 | spd_rs05995	*pyrR*	bifunctional pyrimidine regulatory protein PyrR/ uracil phospho-ribosyltransferase (EC:2.4.2.9)	1.87	s.	1.29	n.s.	**1.64**	**s.**	1.21	n.s.
spd_1226 | spd_rs06470	namex 1289	amino acid ABC transporter amino acid-binding protein	-1.07	n.s.	-1.18	n.s.	**4.50**	**s.**	3.73	s.
spd_1357 | spd_rs07140	*aliB*	oligopeptide ABC transporter oligopeptide-binding protein AliB	-1.11	0.57	-1.06	n.s.	**N/A**	**off**	N/A	off
spd_1518 | spd_rs08005	namex 1595	transcriptional activator, Rgg/GadR/MutR family protein	-1.19	n.s.	-1.03	n.s.	**-2.16**	**s.**	1.05	n.s.
spd_1607 | spd_rs08500	namex 1693	ABC transporter permease	N/A	N/A	N/A	N/A	**N/A**	**on**	N/A	on
spd_1608 | spd_rs08505	namex 1694	ABC transporter ATP-binding protein	1.22	n.s.	-1.07	n.s.	**32.20**	**s.**	54.44	s.

spd_1609 | spd_rs08510	namex 1695	ABC transporter substrate-binding protein	1.54	n.s.	1.17	n.s.	**38.40**	**s.**	107.54	s.
spd_1610 | spd_rs08515	namex 1696	hypothetical protein	1.37	n.s.	1.00	n.s.	**24.62**	**s.**	43.93	s.
spd_1613 | spd_rs08525	*galT-1*	galactose-1-phosphate uridylyltransferase (EC:2.7.7.12)	N/A	N/A	N/A	N/A	**N/A**	**on**	N/A	on
spd_1617 | spd_rs08545	namex 1702	cell wall surface anchor family protein	N/A	N/A	N/A	N/A	**N/A**	**on**	N/A	on
spd_1628 | spd_rs08605	*xpt*	xanthine phospho-ribosyltransferase (EC:2.4.2.22)	-1.55	n.s.	1.11	n.s.	**-2.41**	**s.**	-1.47	n.s.
spd_1667 | spd_rs08820	*amiF*	oligopeptide ABC transporter ATP-binding protein AmiF	-1.53	s.	-1.50	s.	**-1.59**	**s.**	-1.27	s.
spd_1668 | spd_rs08825	*amiE*	oligopeptide ABC transporter ATP-binding protein AmiE	-1.82	s.	-1.95	s.	**-1.92**	**s.**	-1.74	s.
spd_1669 | spd_rs08830	*amiD*	oligopeptide ABC transporter permease AmiD	-2.92	s.	-2.93	s.	**-2.04**	**s.**	-1.97	s.
spd_1670 | spd_rs08835	*amiC*	oligopeptide ABC transporter permease AmiC	-2.83	s.	-3.16	s.	**-2.42**	**s.**	-2.43	s.
spd_1671 | spd_rs08840	*amiA*	oligopeptide ABC transporter oligopeptide-binding protein AmiA	-959.64	N/A	N/A	off	**-2036.0**	**off**	N/A	off
spd_1874 | spd_rs09880	namex 1969	LysM domain-containing protein	1.19	n.s.	-1.00	n.s.	**1.63**	**s.**	1.13	n.s.

[a] significance level or on-off effect (s. – significant with $p \leq 0.05$; n.s. – not significant with $p > 0.05$; N/A – p-value not available; on – on-off effect: quantified only in mutant; off – on-off effect: not quantified in mutant)
Because of missing intensity values in the proteomics data set, not all fold change values could be calculated. Those are indicated by „N/A“.

d. Regulated proteins (n=50) in the comparison of *S.p.* D39Δ*aliB* strain and *S.p.* D39 wild-type strain in mid-exponential growth phase.

			**Δ*comDE* vs. wt**				**Δ*aliB* vs. wt**			
			**early exp. growth phase**		**mid-exp. growth phase**		**early exp. growth phase**		**mid-exp. growth phase**	
**identifier**	**gene**	**description**	**fold change**	**sign.[a]**	**fold change**	**sign.[a]**	**fold change**	**sign.[a]**	**fold change**	**sign.[a]**
spd_0005 | spd_rs00025	*pth*	peptidyl-tRNA hydrolase (EC:3.1.1.29)	N/A	N/A	N/A	N/A	N/A	N/A	**N/A**	**on**
spd_0109 | spd_rs00570	namex 114	amino acid ABC transporter periplasmic amino acid-binding protein	N/A	N/A	N/A	N/A	363.52	on	**N/A**	**on**
spd_0110 | spd_rs00575	*argG*	argininosuccinate synthase (EC:6.3.4.5)	N/A	N/A	N/A	N/A	N/A	on	**N/A**	**on**
spd_0111 | spd_rs00580	*argH*	argininosuccinate lyase (EC:4.3.2.1)	N/A	N/A	N/A	N/A	N/A	on	**N/A**	**on**
spd_0115 | spd_rs00605	namex 121	hypothetical protein	-2.70	off	1.01	n.s.	N/A	off	**N/A**	**off**
spd_0200 | spd_rs01080	*rplP*	5S ribosomal protein L16	1.23	n.s.	-1.09	n.s.	1.01	n.s.	**1.63**	**s.**
spd_0235 | spd_rs01260	*pfl*	pyruvate formate-lyase (EC:2.3.1.54)	1.22	N/A	N/A	N/A	N/A	N/A	**N/A**	**on**
spd_0251 | spd_rs01345	*rpsL*	3S ribosomal protein S12	-1.03	n.s.	-1.05	n.s.	1.14	n.s.	**1.89**	**s.**
spd_0308 | spd_rs01645	*clpL*	ATP-dependent Clp protease, ATP-binding subunit	1.69	s.	1.54	s.	1.76	s.	**2.72**	**s.**
spd_0322 | spd_rs01715	*cps2G*	glycoside hydrolase family protein (EC:2.4.1.-)	-1.05	n.s.	1.00	n.s.	-1.15	n.s.	**1.51**	**s.**
spd_0402 | spd_rs02140	namex 427	hypothetical protein	1.09	N/A	1.41	n.s.	-1.43	N/A	**2.39**	**s.**
spd_0430 | spd_rs02290	*trkA*	potassium transporter peripheral membrane protein	N/A	N/A	-1.07	n.s.	N/A	N/A	**1.68**	**s.**
spd_0453 | spd_rs02405	*hsdS*	type I restriction-modification system subunit S	N/A	N/A	1.25	n.s.	-1.41	N/A	**2.13**	**s.**
spd_0466 | spd_rs02480	namex 495	hypothetical protein	N/A	N/A	N/A	N/A	N/A	N/A	**N/A**	**on**
spd_0587 | spd_rs03145	namex 627	short chain dehydrogenase/ reductase family oxidoreductase	N/A	N/A	-1.07	n.s.	N/A	N/A	**1.60**	**s.**
spd_0661 | spd_rs03520	*exp5*	PTS system transporter subunit IIABC (EC:2.7.1.69)	1.02	n.s.	-1.72	n.s.	2.14	s.	**2.39**	**s.**
spd_0697 | spd_rs03695	namex 736	GNAT family acetyltransferase	N/A	N/A	N/A	on	N/A	N/A	**N/A**	**on**
spd_0719 | spd_rs03805	namex 758	amino acid ABC transporter permease	1.04	n.s.	-1.29	n.s.	1.91	s.	**1.76**	**s.**
spd_0721 | spd_rs03815	*folD*	bifunctional 5,1-methylene-tetrahydro-folate dehydrogenase/ 5,1-methylene-tetra-hydrofolate cyclo-hydrolase	-1.08	n.s.	-1.13	n.s.	1.76	s.	**1.82**	**s.**
spd_0814 | spd_rs04325	namex 861	agmatine deiminase (EC:3.5.3.12)	N/A	N/A	N/A	N/A	-1.08	N/A	**N/A**	**on**
spd_0960 | spd_rs05115	*cpoA*	glycosyl transferase CpoA (EC:2.4.1.-)	N/A	N/A	1.52	n.s.	N/A	N/A	**1.63**	**s.**
spd_0968 | spd_rs05165	namex 1029	GNAT family acetyltransferase	N/A	N/A	N/A	N/A	N/A	N/A	**N/A**	**on**
spd_1020 | spd_rs05435	*rnhB*	ribonuclease HII (EC:3.1.26.4)	N/A	N/A	N/A	N/A	N/A	N/A	**N/A**	**on**
spd_1171 | spd_rs06190	namex 1233	hypothetical protein	N/A	N/A	N/A	N/A	N/A	N/A	**N/A**	**on**
spd_1194 | spd_rs06305	*thrB*	homoserine kinase (EC:2.7.1.39)	1.17	n.s.	1.10	n.s.	1.01	n.s.	**1.66**	**s.**

spd_1226 | spd_rs06470	namex 1289	amino acid ABC transporter amino acid-binding protein	-1.07	n.s.	-1.18	n.s.	4.50	s.	**3.73**	**s.**
spd_1245 | spd_rs06565	*rpsU*	3S ribosomal protein S21	1.10	n.s.	1.18	n.s.	1.10	n.s.	**1.61**	**s.**
spd_1311 | spd_rs06890	namex 1373	oxidoreductase, Gfo/Idh/MocA family protein	1.24	n.s.	1.14	n.s.	1.13	n.s.	**1.72**	**s.**
spd_1349 | spd_rs07095	*murC*	UDP-N-acetylmuramate--L-alanine ligase (EC:6.3.2.8)	1.03	n.s.	1.16	n.s.	-1.04	n.s.	**1.55**	**s.**
spd_1357 | spd_rs07140	*aliB*	oligopeptide ABC transporter oligopeptide-binding protein AliB	-1.11	n.s.	-1.06	n.s.	N/A	off	**N/A**	**off**
spd_1439 | spd_rs07580	*rpsO*	3S ribosomal protein S15	1.46	n.s.	1.49	n.s.	-1.05	n.s.	**5.08**	**s.**
spd_1514 | spd_rs07985	namex 1591	ABC transporter ATP-binding protein	N/A	N/A	-1.09	n.s.	N/A	N/A	**N/A**	**off**
spd_1516 | spd_rs07995	namex 1593	hypothetical protein	N/A	N/A	-1.52	N/A	N/A	N/A	**N/A**	**off**
spd_1559 | spd_rs08230	namex 1640	GTP-binding protein YqeH	1.13	n.s.	1.11	n.s.	-1.06	n.s.	**1.73**	**s.**
spd_1607 | spd_rs08500	namex 1693	ABC transporter permease	N/A	N/A	N/A	N/A	N/A	on	**N/A**	**on**
spd_1608 | spd_rs08505	namex 1694	ABC transporter ATP-binding protein	1.22	n.s.	-1.07	n.s.	32.20	s.	**54.44**	**s.**
spd_1609 | spd_rs08510	namex 1695	ABC transporter substrate-binding protein	1.54	n.s.	1.17	n.s.	38.40	s.	**107.54**	**s.**
spd_1610 | spd_rs08515	namex 1696	hypothetical protein	1.37	n.s.	1.00	n.s.	24.62	s.	**43.93**	**s.**
spd_1613 | spd_rs08525	*galT-1*	galactose-1-phosphate uridylyltransferase (EC:2.7.7.12)	N/A	N/A	N/A	N/A	N/A	on	**N/A**	**on**
spd_1617 | spd_rs08545	namex 1702	cell wall surface anchor family protein	N/A	N/A	N/A	N/A	N/A	on	**N/A**	**on**
spd_1658 | spd_rs08770	namex 1747	hypothetical protein	N/A	N/A	N/A	N/A	N/A	N/A	**N/A**	**on**
spd_1668 | spd_rs08825	*amiE*	oligopeptide ABC transporter ATP-binding protein AmiE	-1.82	s.	-1.95	s.	-1.92	s.	**-1.74**	**s.**
spd_1669 | spd_rs08830	*amiD*	oligopeptide ABC transporter permease AmiD	-2.92	s.	-2.93	s.	-2.04	s.	**-1.97**	**s.**
spd_1670 | spd_rs08835	*amiC*	oligopeptide ABC transporter permease AmiC	-2.83	s.	-3.16	s.	-2.42	s.	**-2.43**	**s.**
spd_1671 | spd_rs08840	*amiA*	oligopeptide ABC transporter oligopeptide-binding protein AmiA	-959.64	N/A	N/A	off	-2036.0	off	**N/A**	**off**
spd_1729 | spd_rs09140	namex 1821	hypothetical protein	1.23	s.	1.03	n.s.	-1.04	n.s.	**1.78**	**s.**
spd_1745 | spd_rs09230	namex 1839	transcriptional regulator PlcR	N/A	N/A	N/A	N/A	N/A	N/A	**N/A**	**on**
spd_1784 | spd_rs09430	namex 1879	ABC transporter ATP-binding protein	-1.70	N/A	N/A	N/A	-1.86	N/A	**N/A**	**on**
spd_1943 | spd_rs10230	namex 2038	hypothetical protein	1.25	N/A	-1.03	N/A	1.32	N/A	**1.70**	**s.**
spd_2025 | spd_rs10630	namex 2118	ABC transporter substrate-binding protein	-1.01	n.s.	1.13	n.s.	-1.14	n.s.	**1.60**	**s.**
spd_2025 | spd_rs10630	namex 2118	ABC transporter substrate-binding protein	-1.01	n.s.	1.13	n.s.	-1.14	n.s.	**1.60**	**s.**

[a] significance level or on-off effect (s. – significant with p ≤ 0.05; n.s. – not significant with p > 0.05; N/A – p-value not available; on – on-off effect: quantified only in mutant; off – on-off effect: not quantified in mutant)
Because of missing intensity values in the proteomics data set, not all fold change values could be calculated. Those are indicated by „N/A“.
