# Supplementary material for: In vivo proteomics identifies the competence regulon and AliB oligopeptide transporter as pathogenic factors in pneumococcal meningitis
Source: PLoS Pathog. 2019 Jul 29;15(7):e1007987. doi: 10.1371/journal.ppat.1007987 (PMC6687184; doi:10.1371/journal.ppat.1007987)
Supplement: S2 Table — (PDF) [file ppat.1007987.s005.pdf]

Table S2 Strain and plasmid list

Strains or plasmids	Serotype and relevant Genotype	Resistance[a]	Source or Reference
Streptococcus pneumoniae			
D39	2		NCTC7466
TIGR4	4		Tetellin et al., 2001
R6	2		Ottolenghi and Hotchkiss, 1962
PN111	D39Δ*cps*	Km^r	Rennemeier *et al*., 2007
PN586	D39Δ*aliB*	Cm^r	this study
PN502	D39Δ*comDE*	Km^r	this study
PN640	D39Δ*aliB*Δ*comDE*	Cm^r, Km^r	this study
PN419	D39Δ*cps*Δ*ply*	Km^r, Cm^r	this study
Escherichia coli			
DH5α	[*Δ* (*lac*) *U169 endA1 gyrA46 hsdR17 ϕ80Δ* (*lacZ*) *M15 recA1 relA1 supE44 thi-1*] host strain for construction of recombinant plasmids	None	Novagen
BL21 (DE3)	Host strain for specific protein expression		Invitrogen
**Plasmids**			
pTP1	pET28 derivative expression vector with a histidin fusion tag and TEV protease cleavage site	Km^r, Erm^r	Saleh *et al*., 2013
pET1033	pTP1 derivative expressing AliB from amino acid 26 to 652	Km^r	this study
pSP72	cloning vector	Amp^r	Promega
p1037	pSP72 derivative with DNA fragment of *aliB* interrupted by *cat* resistance gene	Amp^r, Cm^r	this study
pKK2	pASK-IBA5 derivative expression pneumolysin	Amp^r	this study

[a]Amp, ampicillin; Km, kanamycin; Cm, chloramphenicol ; r, resistant
