# Supplementary material for: In vivo proteomics identifies the competence regulon and AliB oligopeptide transporter as pathogenic factors in pneumococcal meningitis
Source: PLoS Pathog. 2019 Jul 29;15(7):e1007987. doi: 10.1371/journal.ppat.1007987 (PMC6687184; doi:10.1371/journal.ppat.1007987)
Supplement: S3 Table — (PDF) [file ppat.1007987.s006.pdf]

Table S3 Primer list

**primer purpose**	**primer**	**sequence (5'-3')**
**mutagenesis**		
amplification of aliB	aliB_1276	5' -GCGT**GCTAGC**TCAAAAACCTATAACTATGTT- 3'
	aliB_1277	5' -GCGC**GAGCTC**TTATTTGACATGTTTTGCC- 3'
amplification of comDE	comDE_1224	5' -GGGATTCCCCTACCATAAATAGAAAATTGACTTT- 3'
	comDE_1225	5' -GATATGATTGAGCACTATCAAAGCAAGCTATTCC- 3'
	comC_fwd	5'- GCTAAGTTTGAAATGATTGAGTTATCAGATG-3'
	comD_kan_rev2	5'-CCATTTCCTCTGGAATAGGCGTCGACGCGCCCAAATC<br>CAAATAAATCCATTAC-3'
	comD_kan_fwd2	5'-GTAATGGATTTATTTGGATTTGGGCGCGTCGACGCCTA<br>TTCCAGAGGAAATGG-3'
	comE_kan_fwd2	5'-CCGCAACTGTCCATACTCTGATGGGATATTTTAGAG<br>AAAAAATCTC-3'
	comE_kan_rev2	5'-GAGATTTTTTCTCTAAAATATCCCATCAGAGTAT<br>GGACAGTTGCGG-3'
	comE_rev	5'-GTTTACAAGGAGGAAATATGCAAGAAAG-3'
**recombinant protein production**		
spd_1357 (D39)	aliB_1288	5' -GCGT**GCTAGC**GGAAATTCTAGCACTGCATC-3'
	aliB_1277	5' -GCGC**GAGCTC**TTATTTGACATGTTTTGCC-3'
sp_1923 (TIGR4)	ply_370	5'-CG**GGATCC**GCAAATAAAGCAGTAAATGAC-3
	ply_371	5'-GC**GGTACC**CTAGTCATTTTCTACCTGAG-3'

Restriction sites are **bold**; pneumococcal sequences for overlap PCR are underlined. ply: pneumolysin
