# Supplementary material for: In vivo proteomics identifies the competence regulon and AliB oligopeptide transporter as pathogenic factors in pneumococcal meningitis
Source: PLoS Pathog. 2019 Jul 29;15(7):e1007987. doi: 10.1371/journal.ppat.1007987 (PMC6687184; doi:10.1371/journal.ppat.1007987)
Supplement: S4 Table — (PDF) [file ppat.1007987.s007.pdf]

Table S4 LC-MS/MS settings for analysis of samples of *in vitro* cultivated *S. pneumoniae* D39 wild-type and *S.p.* D39Δ*comDE* and *S.p.* D39Δ*aliB* mutants.

a. reversed phase liquid chromatography (RPLC)

instrument	Ultimate 3000 RSLC (Dionex, Idstein, Germany)
trap column	75 µm inner diameter, packed with 3 µm C18 particles (Acclaim PepMap100, Thermo Scientific)
analytical column	Accucore 150-C18, (Thermo Fisher Scientific),<br>25 cm x 75 µm, 2.6 µm C18 particles, 150 Å pore size
buffer system	binary buffer system consisting of 0.1% acetic acid (buffer A) and 0.1% acetic acid in 100% ACN in (buffer B)
flow rate	300 nl/min
gradient	linear gradient of buffer B from 5% up to 25%<br>1 – 2 min: increase from 2% to 5%<br>2 – 10 min: 5%<br>10 – 130 min: increase from 5% to 25%<br>130 – 135 min: increase from 25% to 40%<br>135 – 137 min: increase from 40% to 90%<br>137 – 142 min: 90%<br>142 – 145 min: decrease from 90% to 2%<br>145 – 160 min: 2%
gradient duration	120 min (in total 160 min)
column oven temperature	40°C

b. mass spectrometry (MS)

instrument	Q Exactive mass spectrometer (Thermo Scientific)
electrospray	via TriVersa NanoMate (Advion Biosciences, Norwich, UK)
operation mode	data-dependent
**full MS** resolution	70,000
**full MS** AGC target	3e6
**full MS** maximum ion injection time	120 ms
**full MS** scan range	300 to 1,650 m/z
**dd-MS2**resolution	17,500
**dd-MS2**AGC target	2e5
**dd-MS2** selection for MS/MS	10 most abundant isotope patterns with charge ≥ 2 from the survey scan
**dd-MS2** maximum ion injection time	120 ms
isolation window	3 *m/z*
dissociation mode	higher energy collisional dissociation (HCD)
normalized collision energy	27.5%
dynamic exclusion	30 s
MS/MS operation mode	centroid mode, peptide match: preferred
charge exclusion	1, > 6
